# Effect of Rare-Earth Element Microdoping on Ti–6Al–7Nb Alloys for Biomedical Applications: Materials Characterization and In Vivo Biocompatibility Tests

**DOI:** 10.3390/ma19040709

**Published:** 2026-02-12

**Authors:** Alexander Anokhin, Andrey Kirsankin, Elena Ermakova, Maria Chuvikina, Alexander Luk’yanov, Svetlana Strelnikova, Elena Kukueva, Nataliya Kononovich, Konstantin Kravchuk, Joydip Joardar

**Affiliations:** 1 Baikov Institute of Metallurgy and Materials Science, Russian Academy of Sciences (IMET RAS), Leninsky Prospect 49, Moscow 119334, Russia; akirsankin@imet.ac.ru (A.K.); eadrshina@imet.ac.ru (E.E.); mchuvikina@imet.ac.ru (M.C.); alukyanov@imet.ac.ru (A.L.); sstrelnikova@imet.ac.ru (S.S.); 2 National Research Center Kurchatov Institute, 1 Akademika Kurchatov Square, Moscow 123182, Russia; elena.kukueva@gmail.com; 3 National Ilizarov Medical Research Centre for Traumatology and Ortopaedics, 6 Maria Ulyanova Street, Kurgan 640021, Russia; n.a.kononovich@mail.ru; 4 National Research Center Kurchatov Institute—TISNCM, 7A Tsentralnaya Street, Troitsk, Moscow 108840, Russia; kskrav@gmail.com; 5 International Advanced Research Centre for Powder Metallurgy and New Materials (ARCI), Balapur Post Office, Hyderabad 500005, Telangana, India; joydip@arci.res.in

**Keywords:** REEs, rare-earth elements, lanthanum, La, cerium, Ce, yttrium, Y, microalloying, microdoping, titanium, Ti, titanium alloys, Ti-based alloys, TiAlNb, Ti6Al7Nb, alpha titanium alloy, beta titanium alloy, biocompatible alloys, material characterization, personal implants, biocompatibility, biocompatible tests, in vivo

## Abstract

The paper focuses on materials characterization and in vivo biocompatibility tests of Ti–6Al–7Nb–0.3REE wt.% alloys (REEs—Y, Ce, La) for use as a promising material to produce personalized medical implants and shed light on possible toxicity effects of REE alloy microdoping. All alloys were produced by the electric arc melting method and characterized by scanning electron microscopy (SEM), optical microscopy (OM), energy-dispersive X-ray spectroscopy analysis (EDX), X-ray diffraction (XRD), true density analysis, micro- and nanoindentation methods, and reducing/oxidation melting techniques. True density of alloys increased in the following order: Ti−6Al−7Nb−0.3Y (4.4563 ± 0.1075 g/cm^3^) < Ti−6Al−7Nb−0.3Ce (4.7255 ± 0.2853 g/cm^3^) < Ti−6Al−7Nb−0.3La (4.8019 ± 0.0111 g/cm^3^). XRD analysis indicated that Ti–6Al–7Nb–0.3Y alloy consisted of single α–Ti phase in comparison with Ti–6Al–7Nb–0.3La (α–Ti to β–Ti = 82 to 18) and Ti–6Al–7Nb–0.3Ce (α–Ti to β–Ti = 90.5 to 9.5). The single-phase Ti–6Al–7Nb–0.3Y alloy had the finest α–Ti phase crystallites (22.32 nm); the larger α–Ti crystallites in the dual-phase Ti–6Al–7Nb–0.3Ce and Ti–6Al–7Nb–0.3La (30.77 nm and 29.83 nm, respectively) suggested the presence of the β–Ti phase (23.34 nm and 25.61 nm, respectively). REE microdoping of alloys changed the lattice volume (∆V): α–Ti phase—0.269% for Ti–6Al–7Nb–0.3Y, 1.799% for Ti–6Al–7Nb–0.3Ce, 0.595% for Ti–6Al–7Nb–0.3La; and β–Ti phase—0.334% for Ti–6Al–7Nb–0.3Ce, 0.670% for Ti–6Al–7Nb–0.3La. Nanohardness (H) and elastic modulus (E) increased in the following order: Ti−6Al−7Nb−0.3La (4.01 GPa and 135 GPa, respectively) < Ti−6Al−7Nb−0.3Y (4.39 GPa and 137 GPa, respectively) < Ti−6Al−7Nb−0.3Ce (4.67 GPa and 146 GPa, respectively). In vivo tests were conducted using 46 sexually mature male Wistar rats by means of skin implantation of samples with d = 11 mm and h = 1 mm. Our research shows that Ti–6Al–7Nb–0.3La alloy (Group 2) and Ti–6Al–7Nb–0.3Ce alloy (Group 3) induced sustained hepatotoxic and nephrotoxic effects. Ti–6Al–7Nb–0.3Y alloy induced a slight local inflammatory response; however, serum biochemical analysis suggested this effect was compensated.

## 1. Introduction

Titanium and its alloys play a predominant role as structural biomaterials in reconstructive surgery, especially orthopedics and maxillofacial surgery [1,2,3,4,5,6,7,8,9,10,11,12]. Based on the fact that Pure Titanium and its alloys undoubtedly have excellent properties as biomaterials for surgical implants, current scientific work [13,14,15,16,17,18,19,20] focuses on increasing the efficiency of these materials by introducing optimal alloying components. This allows for maintaining a high level of biocompatibility [21,22,23,24,25,26,27,28,29,30,31,32,33,34,35,36,37,38,39]. Metals such as tantalum, Niobium, zirconium and, in certain situations, molybdenum are suitable candidates for such modification [14,16,40,41,42,43].

The addition of alloying elements such as Nb, Si, Mo, Ta, W, Fe, Cr and some rare-earth metals can stabilize the β phase [40,44,45,46,47,48,49,50,51]. TiAl-based alloys with 40–50 at.% of Nb are exceptionally rare in the literature because they would drastically alter the fundamental phase constitution and all properties of the alloys. Mechanical properties are improved by the addition of a high amount of Nb to TiAl-based alloys, causing an increase in the Nb content in Ti–Al–Nb alloys from 40 to 50 at.% [52,53,54,55]. Al promotes the formation of γ–TiAl and σ–Nb_2_Al phases in the structure of the material, improving its properties at high temperatures. Previous research has established that modifying the composition of γ + σ phases within the structure enhanced the fracture resistance of Ti-, Al-, and Nb-based composites with elevated σ-phase content [52,53,54,55,56,57,58,59,60]. However, the main research focuses on a lower Nb range of 5–10 wt.% because of the wide use of those alloys for medical applications, such as Ti–6Al–7Nb wt.% [36,61,62,63,64,65,66,67,68,69].

Ti-based alloys have the effect of a spontaneous surface reaction called auto-oxidation; it is not merely a beneficial passivation mechanism but a critical, continuous process that extends into the bulk material at elevated temperatures or under mechanical stress, dictating long-term stability and functionality [70,71,72,73]. On the one hand, it is the genesis of the crucial passive film. On the other hand, it can drive the internal oxidation of reactive elements like Al and Nb, leading to the precipitation of stable oxides (e.g., Al_2_O_3_, Nb_2_O_5_) and nitrides within the matrix or at grain boundaries [74,75]. For Ti–Al–Nb alloys that have been used in biomedical implants, auto-oxidation is a double-edged sword. The stable surface oxide is essential for biocompatibility and osseointegration. Yet, the particulate debris generated from the brittle fracture of an oxygen-embrittled microstructure or from the spallation of a thick oxide scale can trigger a chronic foreign-body response, leading to inflammation, periprosthetic osteolysis, and, ultimately, implant failure [76,77,78].

Other researchers confirmed that small additions of rare-earth elements make it possible to control the structure of Aluminum and other alloys due to formation of secondary intermetallic phases [46,47,79]. The average grain size decreases with increasing Ce content, and the grain gradually passes from a large lamellar structure to a two-phase microstructure [79]. The addition of Yttrium has a positive effect on the structural stability of alloys, which reduces their liquation heterogeneity and prevents the formation of harmful structural components [47,80]. It promotes the removal of interstitial oxygen in the α and γ–Ti phases [3]. Dispersed oxide particles in the microstructure of the alloys containing La decrease dynamic recrystallization [81,82].

Bartáková, S. et.al. of [83] focused on 34 different compositions of new Titanium β-alloys with different amounts of Niobium (Nb) and other metals such as molybdenum (Mo), tantalum (Ta), and Vanadium (V), and they carried out numerous biological tests to evaluate the biocompatibility of Ti-based β-alloys. A table with data on the biocompatibility of the main alloying elements, Ti, Al, Nb, V, Zr, Mo, Ta, Fe, Cu, and Zn, was considered in previous work [3,14,40,41,46,48,50,51,84,85,86,87]. Ti-based β-alloys possess low elastic moduli, between 40 and 80 GPa. This is comparable to the elastic modulus of bone, thus helping to prevent bone absorption, which could otherwise lead to implant loosening or refracture of the bone following removal [82,88].

It is widely known that rare-earth elements (REEs) are a group of metals that include Lanthanum (La), 14 lanthanides, Yttrium (Y), and scandium (Sc). However, the contradictions between adverse (e.g., toxicological and ecotoxicological) and beneficial effects associated with REEs have not been fully discussed. There are a number of reviews that address both the toxic effects on the body [89,90] and the antioxidant effects associated with REEs in the treatment of many diseases [90,91,92,93], as well as the use of REEs as alternative safe feed additives used to stimulate growth and performance of animals (Table 1) [90].

Despite the toxicity of REEs when used as solutions, there are proven studies showing that alloy doping with REEs does not have a toxic effect. An experimental study on a specific dental alloy with composition Ti–Fe–Mo–Mn–Nb–Zr, doped with 0.3% wt. of Cerium, shows no cytotoxicity observed in acute toxicity, hemolytic, and MTT assays. It was confirmed that 0.3 wt. % of Cerium microdoping is innocuous within the concentration range of this study [94]. Deng, Z. et.al. of [95] reported that hydroxyapatite coating on Titanium with Lanthanum has comparable cytotoxicity in comparison with La-free hydroxyapatite coatings, but it provides no primary toxicity data. A heavy dosage of La and Ce has severe toxicity towards MC3T3–E1 murine calvarial pre-osteoblasts [96], which is contrary to results of Willbold, E. et.al. of [15], who reported no systematic or local cytotoxicological effects during other in vivo and in vitro tests. Charbgoo, F. et.al. of [97] reported that the ability of Cerium to switch between oxidation states (Ce^3+^/Ce^4+^) affects antioxidant and anti-inflammatory properties. Cerium can help mitigate inflammatory responses as well, which sometimes hinders implant integration [98,99].

The chemical composition requirements for Ti–6Al–7Nb alloy were determined by ASTM F1295–24 and ISO 5832–11:2024 standards, having little differences between chemical requirements such as impurities of oxygen (0.2 wt. % ± 0.02), carbon (0.08 wt. % ± 0.02), nitrogen (0.05 wt. % ± 0.02), hydrogen (0.009 wt. % ± 0.002), Iron (0.25 wt. % ± 0.10), and cobalt (0.10 wt. % ± 0.02); other elements need not be reported unless the concentration level is greater than 0.1 wt. % each or 0.4 wt. % total [100,101].

Thus, on the one hand, existing data on the in vitro and in vivo biocompatibility of REE-containing alloys are very controversial, partially at low concentrations (≤0.3 wt. %). On the other hand, current standards ASTM F1295–24 and ISO 5832–11:2024 for Ti–6Al–7Nb alloys allow the accumulation of REEs in an amount of no more than 0.4 wt. %, but at the same time, the content of each REE does not exceed 0.1 wt. %. There are no specific limits considering type of REEs (Table 2).

The microstructure, phase composition, and mechanical properties of Ti-based alloys fundamentally play an important role in interaction with different tissues. While there is no direct detailed research related to subcutaneous implantation of Ti–Al–Nb alloys, the underlying principles are well-documented for bone–implant scenarios in different in vitro and in vivo experiments. In vivo experiments with β–Ti alloy (Ti–35Nb–7Zr–5Ta)_98_Si_2_ show significantly better osseointegration than Ti–6Al–4V with a much lower elastic modulus (37 GPa in comparison to 110 GPa, respectively) [102]. Also, in vitro studies show that Si doping into Ti–Nb–Zr alloy significantly increases the corrosion resistance in simulated inflammatory-containing peroxide and lactate environments due to stability of the passive oxide layer [38]. Biguetti, C.C. et.al. of [103] proved in an in vivo study in mice that a direct causal relationship, such as intentionally induced corrosion of Pure Titanium and Ti–6Al–4V implants, leads to a persistent chronic inflammatory response, impaired healing, and failure of osseointegration.

Jemat A. et.al., Wu Y et.al., Martín-García M. et.al., Bierbaum S. et.al., Quinn J. et.al., Krzywicka M. et.al., Shida T. et.al., Gasik M. et.al., Kunrath M. et.al., Yoda I. et.al., Paulitsch-Fuchs A.H. et.al, Crawford R.J. et.al., Damiati L. et.al., Yeo I.S. et.al., and Braem A. et.al. of [34,104,105,106,107,108,109,110,111,112,113,114,115,116,117] reported that surface roughness is critically important for bone–implant contact and cell proliferation, and it increases removal time; optimal roughness accelerates osseointegration. Sintered porous Titanium HIP prostheses showed superior early bone ingrowth and osseointegration compared to dense, coated materials [118]. A study [119] found that porous Ti–6Al–4V alloy with 75% porosity allows for optimal bone ingrowth. In cavities with a moderately rough inner surface, bone formation (BIC—bone impact contact) is significantly higher than in smooth ones [120]. Roughness increases the risk of bacterial colonization as well, which can lead to infection, inflammation, and local fever. The adhesion of Staphylococcus epidermidis is significantly higher on rougher surfaces (Ra > 0.2 μm).

Oxygen (O) in Ti-based alloys is an α–Ti phase stabilizer that increases strength and reduces ductility. High oxygen content is very critical for oxide layer or non-metallic inclusion formation, which improves fracture toughness and reduces risk of implant debris, but it can cause brittle fracture, generating particulate debris that triggers chronic inflammation [121,122,123,124,125,126]. Nitrogen (N) is strong α–Ti phase stabilizer as well; it dramatically increases strength and hardness but severely embrittles. High levels are likely to lead to implant cracking under cyclic skin movement, causing debris and a sustained foreign-body response [127,128,129,130,131]. Sulfur (S) forms sulfide inclusions (e.g., TiS), which can be stress concentrators and initiate pitting corrosion of the implanted sample; it increases localized corrosion, releasing ions/particles that could provoke local toxicity or granuloma formation [132,133,134]. Phosphorus (P) works as a sulfur (S) inclusion in Ti-based alloys, resulting in brittle intermetallic compound formation. These have effects on potential inclusion-driven local corrosion and adverse tissue reaction, though data is scarce for Ti-based alloys, but it is a rare trace impurity [135,136,137]. Hydrogen is an interstitial impurity and hydrogen embrittlement corrosion (HIC) agent for Ti-based alloys, causing severe loss of ductility and fractured toughness at very low concentrations. There is risk of brittle fracture of the implant under skin that leads to sudden failure and release of large fragments of alloy inside tissues [138,139,140].

REEs refine grains and act as oxygen scavengers due to high oxygen affinity in Ti-based alloys. Lanthanum (La) additive (e.g., ~0.9 wt.% for machinability) forms discrete particles, which slightly decreases mechanical properties and corrosion resistance. In addition, in vitro cytocompatibility can be maintained and overall biocompatibility may remain acceptable if corrosion is controlled [141,142,143]. Cerium (Ce) or Yttrium (Y) are often added to refine grain size, to improve oxidation resistance, and to form oxide dispersions so that CeO_2_ additions refine the β–Ti grain microstructure because dispersed CeO_2_ particles can be centers of nucleation and crystallization. The refinement effect depends on the size and dispersion of the rare-earth oxides [144]. The Yttrium content in Y-Ti alloys influences grain size, resistance against room-temperature oxidation, and gettering performance for oxygen [79,145,146,147,148]. Chromium (Cr) is β–Ti phase stabilizer for all Ti-based alloys that improves hardness and corrosion resistance via a stable Cr_2_O_3_ layer [149,150,151]. Chromium ion release may cause type IV hypersensitivity reactions (potential allergy reaction), leading to persistent dermatitis, eczema, or implant rejection in sensitized individuals [152,153]. Iron (Fe) is common impurity for Ti-based alloys, and it has a β–Ti phase stabilizer effect, which increases strength [154,155,156]. Iron can segregate on grain boundaries, potentially lowering corrosion resistance [156]. Fe-rich phases create galvanic cells, accelerating ion release (Fe, Ti, Al) and accelerating corrosion [156,157,158]. This can lead to metallosis, discoloration of surrounding tissue, and chronic inflammation [157,159,160]. Thus, it is very important to minimize the chronic inflammatory response because the body will wall off any material it deems a persistent threat, forming a fibrous capsule. Elements that promote corrosion (Fe, S inclusions), brittle fracture (high O, N, H), or allergy (Cr) directly fuel this response by continuously releasing ions or particles.

The subcutaneous implantation model is a standard and necessary procedure in preclinical testing of all novel pharmaceuticals, implant materials, and medical devices intended for human use. This model involves the surgical or injectable introduction of material or device samples beneath the skin (or into other tissues) of laboratory animals to evaluate their short- and long-term biocompatibility, toxicity, tissue integration, and effects (e.g., drug release) in vivo.

The method enables the study of both local and systemic organism responses, including inflammatory processes, connective tissue formation, and material resorption. It constitutes an integral component of international testing standards, such as ISO 10993–6, for evaluating novel medical devices. Ultimately, this model is employed to determine the safety of new materials upon contact with human tissues.

Given the controversial data on the biological impact of trace REEs, a systematic screening of their effects is warranted. The aim of the current work is to develop Ti–6Al–7Nb–0.3REE (REEs—Y, Ce, La) novel alloys and shed light on the effect of ≤0.3 wt. % REE microdoping on microstructure, mechanical properties, and phase composition, as well as to study novel alloys’ biocompatibility by means of an in vivo subcutaneous implantation model on Wistar rats because of previously controversial scientific results.

The study is designed to identify potential differences in behavior between Y, Ce, and La (REE) at ≤0.3 wt. % concentration level and to provide foundational data for more targeted future development of REE-modified Ti-based implant materials.

## 2. Materials and Methods

**Smelting of master alloy and Titanium alloy:** Titanium (99.9% purity), Aluminum (99.99% purity), Niobium (99.9% purity), Lanthanum (99.9% purity), Cerium (99.9% purity) and Yttrium (99.9%) were used as a raw materials to produce Aluminum–Rare-Earth Element master alloys (Al–REE) and Ti–6Al–7Nb–0.3REE alloys (REEs—Y, Ce, La). The raw materials were weighed with an accuracy of 0.0001 g on AS 220/C/2 (RADWAG, Radom, Poland). Master alloys Al–10Ce, Al–10La and Al–10Y were placed in a resistance furnace in a graphite crucible to perform microalloying and homogeneous distribution of rare-earth elements (REEs) in the Ti–6Al–7Nb–0.3REE (REEs—Y, Ce, La) alloys [161,162,163,164]. The alloys were smelted by the electric arc melting method in a vacuum using copper cooling molds [165,166,167,168]. All abovementioned initial raw components and master alloys according to composition Ti–Al–Nb–0.3REE (REEs—Y, Ce, La), with total weight 200 g, were subjected to multiple smelting cycles using the arc melting technique. A tungsten electrode with a diameter of 8 mm was used as a non-consumable cathode. Before melting, the chamber was evacuated to a pressure of 7 Pa to remove the oxidative atmosphere and subsequently backfilled with high-purity argon to a pressure of 1.1 atm (absolute). Smelting was conducted using a direct current (DC) power supply, with a stable arc maintained at a voltage of 50 V and a current of 50 A. Each remelting cycle lasted for 90 s to ensure thorough homogenization. To achieve a high level of chemical and structural uniformity, the entire process was repeated for a total of four complete solidification cycles and flipping ingots from one side to another side. Compositional homogeneity of Ti–6Al–7Nb–0.3REE (REEs—Y, La, Ce) alloys was ensured by the intense stirring of the melt via a high-frequency electromagnetic field during remelting and alloying in a vacuum induction melting (VIM) furnace [165,169,170].

**True Density Measurements:** True density of Ti–6Al–7Nb–0.3REE (REEs—Y, Ce, La) alloys was measured using an Ultrapyc 1200e gas pycnometer (Quantachrome Instruments, Boynton Beach, FL, USA) with an accuracy of ±0.03% in a high-purity helium atmosphere of 99.999% with constant gas flow. The cell calibration with volume 10 cm^3^ was performed using 7.0699 cm^3^ stainless–steel spheres. True density was calculated using a built-in computing program based on the Archimedes displacement principle [74,171,172].

**Electron Microscopy (SEM) and Energy-Dispersive X-Ray Spectroscopy (EDS):** SEM and EDS analysis were performed on the polished top sides of samples of Ti–6Al–7Nb–0.3REE (REEs—Y, Ce, La), size 11 mm in diameter and 1 mm in height. Ti–6Al–7Nb–0.3REE (REEs—Y, Ce, La) microstructure was evaluated by SEM images, obtained using a FEI Dual Beam Helios NanoLab system (FEI, Thermo Fisher, Waltham, MA, USA). Cross-sections were produced by a focused ion beam (FIB) technique, employing an FEI Dual-Beam Helios NanoLab system (FEI, Thermo Fisher, Waltham, MA, USA). Elemental analysis was performed with an EDAX system (AMETEK, Mahwah, NJ, USA) [74,173,174,175].

**Analysis of Oxygen, Nitrogen, Hydrogen, Sulfur and Carbon Contents:** The total oxygen, nitrogen and hydrogen content of Ti–6Al–7Nb–0.3REE (REEs—Y, Ce, La) was determined using a TC–600 (LECO Corporation, Saint Joseph, MI, USA) gas analyzer by the method of reducing melting in a nickel capsule in a graphite crucible in a flow of inert carrier gas—helium. Oxygen detection was carried out by measuring the amount of CO and CO_2_ by the infrared absorption method. Nitrogen detection was carried out by thermal conductivity. The total content of carbon and sulfur in Ti–6Al–7Nb–0.3REE (REEs—Y, Ce, La) was determined on a CS–600 (LECO Corporation, Saint Joseph, MI, USA) gas analyzer by the method of oxidative melting in a ceramic crucible in an induction furnace in the presence of a flux—a mixture of metallic tungsten, iron and tin. Carbon and sulfur detection was performed by measuring the amount of released gaseous CO_2_ and SO_2_ by the infrared absorption method [74].

**X-Ray Fluorescence Spectrometry:** Elements analysis of Ti–6Al–7Nb–0.3REE (REEs—Y, Ce, La) alloys was determined on polished samples 11 mm in diameter and 1 mm in height by X-ray fluorescence analysis using a sequential wave-dispersive X-ray fluorescence spectrometer, S8 Tiger Series 2 (Bruker, Karlsruhe, Germany). The spectrometer is equipped with an OEG 95LT X-ray tube with rhodium (Rh) anode with a maximum power of 4 kW and current up to 170 mA, crystal analyzers set, flow-proportional and scintillation detectors, collimators, as well as Al and Cu filters of various thicknesses. The spectrometer is controlled, and spectral data are processed using the Spectra Plus software package, which includes Quant–Express software version 4.2.1. for semi-quantitative (standard-free) express analysis of samples of unknown composition [74].

**X-Ray Phase Analysis (XRD):** X-ray phase analysis (XRD) registration of X-ray diffraction spectra of Ti–6Al–7Nb–0.3REE (REEs—Y, Ce, La) alloys was carried out on the X-ray diffractometer Ultima IV (Rigaku, Tokyo, Japan), with a vertical goniometer and high-speed semiconductor detector “D/teX”, with an Ni filter on the primary beam, in CuKα—radiation was in the range of angles of 2 theta from 9 to 100 degrees, with a shooting step of 0.02 degrees. The qualitative phase and analysis of samples was performed in the PDF-4+ package and Sleve + 2020 software package using the ICDD 2018 and ICSD 2021 database. Assuming the crystallites to be spherical, the coherent scattering region (CSR) dimensions were estimated by applying the Scherrer formula to the X-ray diffraction peak broadening, in accordance with the principles of the Debye–Scherrer and Bragg equations. Quantitative phase analysis (α/β ratio) was performed using the Reference Intensity Ratio (RIR) method. The intensities of the (100) peak for α−Ti and the (110) peak for β-Ti were measured and corrected using the RIR values from the ICDD 2018 database to account for texture effects. The reported phase fractions are derived from these corrected intensities [74,176].

**Hardness (H) and Elastic Modulus Measurements (E)**: Hardness, elastic modulus and ratio of elastic work to the total work of indentation (η_it_) of Ti–6Al–7Nb–0.3REE (REEs—Y, Ce, La) alloys were measured according to ISO 14577 metallic materials—instrumented indentation test for hardness and material parameters [177,178,179,180,181,182]. All samples were hot-poured into epoxy resin and then polished to a roughness at least 100 nm. A triangle Berkovich pyramid was used to do nanoindentation. A series of no fewer than twenty indentations was made for each applied load, with adjacent measurement points separated by at least five times the major dimension of the residual imprint to ensure data integrity. The surface roughness was measured using a Surtronic Neox optical profilometer (Sensofar–Tech, Terrassa, Spain).

**In Vivo Safety Tests** of the Ti–6Al–7Nb–0.3REE (REEs—Y, La, Ce) novel alloys using intravital research methods on the animals recorded the following: body weight (g), overall body temperature (°C) and local body temperature in the implantation area (°C) during the following periods: immediately before the start of the experiment and 3, 7, 14, 21 and 28 days after the operation. The values obtained before the surgery were taken as the norm [183,184,185,186,187].

***Study design:*** The selection of animal species, the number of subjects in each group, and the duration of the study were determined in accordance with information from recognized literature sources in accordance with bioethical principles and guidelines for humane treatment. The sample size was determined with the objective of ensuring sufficiency for the statistical analysis. All research methods employed in this study are readily reproducible. The experimental design was developed with the intention of facilitating replication by other researchers. In vivo experiments were conducted on 46 sexually mature male Wistar rats.

***Sample Size:*** Wistar rats were randomly allocated into four groups: one Control Group for Pure Titanium (*n* = 10) and three experimental groups (*n* = 12 per group) for Ti–6Al–7Nb–0.3Y (Group 1), Ti–6Al–7Nb–0.3La (Group 2), and Ti–6Al–7Nb–0.3Ce (Group 3). The animal model, group sizes, and study duration were selected in accordance with established protocols from the literature for assessing the toxicity of materials containing similar rare-earth elements, which also utilize sexually mature male Wistar rats. Randomization was performed by a blinded staff member prior to surgery. For comparisons between multiple independent groups (e.g., experimental groups vs. control at a given time point), the Kruskal–Wallis test was used. When a significant difference was detected (*p* < 0.05), post hoc pairwise comparisons were conducted using the Dunn test with the Bonferroni correction for multiple comparisons. Published studies in this field typically employ 6 to 10 animals per experimental group or endpoint (e.g., per euthanasia time point). A 28-day observation period, consistent with our experimental design, is a standard duration for such biocompatibility assessments. Furthermore, the sample size was determined to adhere to the bioethical principle of reduction while ensuring adequate statistical power for analysis.

***Inclusion and Exclusion Criteria:*** Inclusion criteria comprised clinically healthy, sexually mature male Wistar rats aged 8–10 months. Exclusion criteria were as follows: clinical signs of any disease, animals of a different sex (females), and animals outside the specified age range. Prior to the study, it was also established that animals would be excluded from the analysis in the event of health deterioration or death unrelated to the experimental procedure. No other exclusion criteria were applied. No animals met the conditions for exclusion during the experimental period. In the Control Group, a total of 50 clinical examinations were performed. Similarly, 50 procedures each were conducted for body weight measurement, overall body temperature recording, and local body temperature measurement in the implantation area. An identical regimen of observations and measurements was carried out in each experimental group, resulting in 60 procedures per type of assessment per group over the study duration.

***Randomization:*** Prior to the experiment, animals were randomly allocated into four groups. To minimize potential confounding factors, stringent standardization procedures were implemented. All surgical interventions were performed by a single surgical team over a two-day period within the same calendar week. Pre- and post-operative housing conditions were uniform: animals were housed in pairs per cage within a single vivarium room where stable temperature and humidity were maintained. Consistency in husbandry and data collection was ensured throughout the study. Cage maintenance, feeding, and watering were performed exclusively by one designated vivarium staff member. All physiological measurements and examinations were conducted by the same researcher. Furthermore, these assessments were consistently performed at identical time points in the morning, prior to the first daily feeding.

***Blinding:*** Animals were randomly allocated into four groups immediately prior to the experiment. This initial allocation was performed by a vivarium staff member who was blinded to the specific implant types designated for each group. This information subsequently remained concealed from the staff member responsible for animal husbandry throughout the study. The definitive random allocation of animals to their specific experimental or control groups was performed by the experimenter immediately prior to the surgical intervention.

***Outcome measures:*** All animals underwent comprehensive clinical examinations, which included assessment of their general appearance, behavioral responses, intensity and nature of motor activity, condition of the fur and skin, and color of the mucous membranes. A visual assessment of the soft tissue condition at the implantation site was performed. Body weight was measured dynamically throughout the experiment using electronic scales TV_A (Massa K, Saint Petersburg, Russia). Core body temperature was recorded with an electronic thermometer DT–622 (AND, Tokyo, Japan) inserted rectally. Measurements continued until an auditory signal confirmed data acquisition was complete. Local body temperature at the implantation site was measured contactlessly using thermal imaging equipment. Significant weight loss or gain can signal the systemic toxicity of the test substance or intervention. Core body temperature was monitored to evaluate the organism’s systemic response to the implantation of test alloy samples, as significant shifts toward hypothermia or hyperthermia may indicate systemic toxic effects or disruption of thermoregulatory processes. This measurement is an essential component of toxicological studies in laboratory animals.

***Statistical Methods:*** Quantitative data were subjected to statistical processing. The initial data and results were collated, corrected, and systematized using Microsoft Office Excel 2016. Statistical analysis was performed with the AtteStat add-in (version 13.1) for Excel (2016, build 16.0.5278.1000). The quantitative data were compiled into variation series. Descriptive statistics were applied to calculate the median (Me) and the first and third quartiles (Q1–Q3). To assess differences between two paired samples, the Wilcoxon signed-rank test (W-test) was employed. For comparisons between experimental groups and the control (independent samples), the Kruskal–Wallis test was used. Differences were considered statistically significant at a significance level of *p* < 0.05.

***Experimental animals:*** The study utilized clinically healthy, sexually mature male Wistar rats aged 8–10 months. All animals were procured from the same specialized laboratory animal breeding facility. No procedures were performed on the animals prior to their arrival at the vivarium.

***Experimental procedures:*** Prior to the commencement of the experiment and at the onset of the procedure, the animals were maintained under conditions that were indistinguishable from those described above, with two animals per cage. The cages housing the animals were situated within a single room of the vivarium, where a uniform temperature regime and air humidity were maintained. Throughout the experiment, cage cleaning, feeding, and water supply were carried out by the same vivarium employee. All research manipulations with animals were performed by the same experimenter. The experimental design involved the administration of examinations at consistent intervals during the morning hours, prior to the initial feeding of the subjects. During the experiment, the recommended temperature regime (24–26 °C) was maintained in the room housing the rats in the vivarium. The animals were maintained on a standard balanced diet, with unrestricted access to water. In order to minimize potential confounding factors, all animals were operated on by one surgical team over two days in the same calendar week. All surgical interventions were performed under operating room conditions. The animals were premedicated with a combination of drugs to suppress consciousness, ensure areflexia, and block pain sensitivity. For the purpose of general anesthesia, drugs were administered at doses calculated in accordance with the subject’s weight (Rometar 2%, 1–2 mg/kg (Bioveta, Ivanovice na Hane, Czech Republic); Zoletil 100, 10–15 mg/kg (Virbac Sante Animale, Carros, France). All examinations were performed at the following time points: immediately prior to the experiment (baseline) and subsequently at weekly intervals on days 7, 14, 21, and 28 post-implantation. All assessments were conducted in the morning hours, prior to the animals’ first daily feeding. All clinical observations and investigative procedures were performed directly within the vivarium room housing the animal cages. To ensure consistency, all observations and measurements were carried out by the same designated vivarium personnel and researchers. Monitoring body weight is a key indicator of overall health status in preclinical animal studies. The experimental model of subcutaneous implantation was selected due to its pivotal role in preclinical trials, wherein samples of materials or devices are surgically or injectionally introduced beneath the skin of animals (or into other tissues) to assess their short-term and long-term biocompatibility, toxicity, integration with tissues, and effects (e.g., drug release) under natural conditions. This method facilitates the examination of the local and systemic reaction of the organism, incorporating processes such as inflammation, connective tissue formation and resorption. It constitutes an integral component of the standards for evaluating the efficacy of novel medical devices. This method is employed to ascertain the safety of new materials for human health when used.

***Ethical statement***: An ethical committee of the RSF № 24–43–02066 project approved to use animals in this study based on statement №34/1 from 22 March 2024. During in vivo biocompatibility tests of novel alloys Ti–6Al–7Nb–0.3REE (REEs—Y, Ce, La), the convention for the Protection of Vertebrate Animals used for Experimental and other Scientific Purposes and the Directive 2010/63/EU of the European Parliament and of the Council of the European Union of 22.09.2010 on the protection of animals used for scientific purposes were used.

## 3. Results

**Figure 1:**
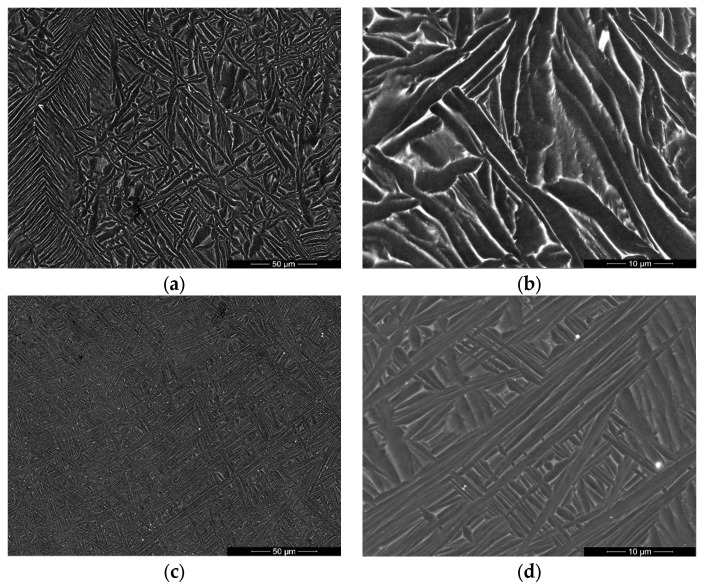

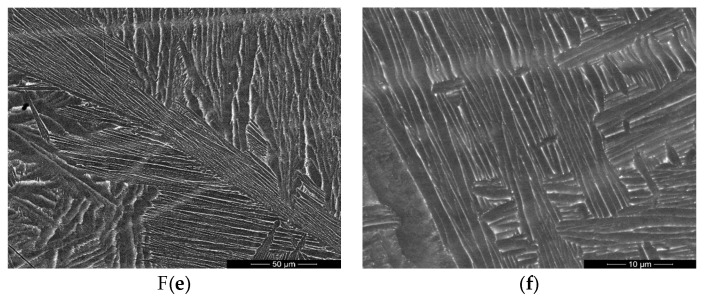
SEM images of Ti–6Al–7Nb–0.3REE (REEs—Y, La, Ce) alloys: (**a**,**b**) Ti–6Al–7Nb–0.3Y alloy microstructure, magnification 500× and 2500×, respectively; (**c**,**d**) Ti–6Al–7Nb–0.3La alloy microstructure, magnification 500× and 2500×, respectively; (**e**,**f**) Ti–6Al–7Nb–0.3Ce alloy microstructure, magnification 500× and 2500×, respectively.

SEM images of Ti–6Al–7Nb–0.3REE (REEs—Y, La, Ce) showed a fine, dispersed, acicular, needle-like pattern, typical for the hexagonal α–Ti phase for Ti–6Al–7Nb–0.3Y alloy (Figure 1a,b), and ultrafine acicular needles were identified, typical of the α–Ti phase, in addition to grain boundaries characteristic of the cubic β–Ti phase for Ti–6Al–7Nb–0.3La alloy (Figure 1c,d) and Ti–6Al–7Nb–0.3Ce alloy (Figure 1e,f). There were no indicated inclusions or other defects of the microstructure.

**Figure 2:**
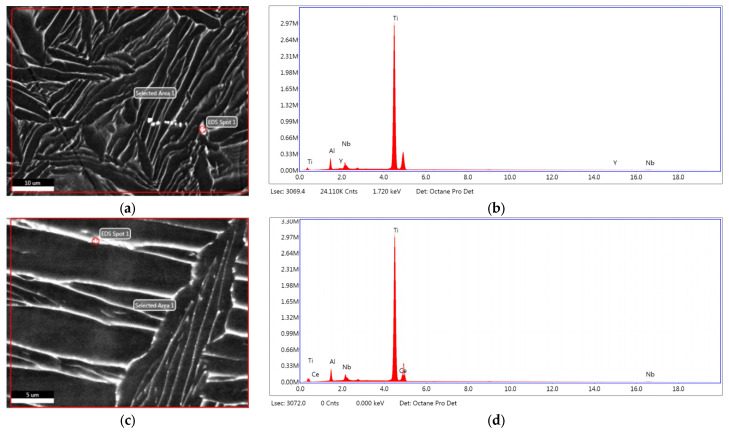

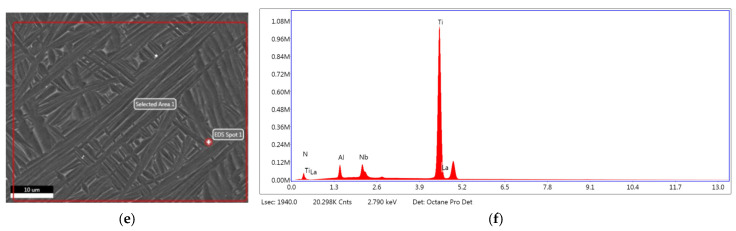
SEM images indicating areas/points of interest and their EDS spectra of Ti–6Al–7Nb–0.3REE (REEs—Y, La, Ce) alloys: (**a**,**b**) Ti–6Al–7Nb–0.3Y alloy, (**c**,**d**) Ti–6Al–7Nb–0.3Ce alloy, (**e**,**f**) Ti–6Al–7Nb–0.3La alloy.

EDS spot and surface analyses were performed on specific areas of interest on the samples’ polished surfaces (selected area 100 μm × 100 μm and 50 μm × 50 μm, and selected spot 1 μm in diameter), reporting the registered elemental composition of Ti, Al, Nb, and Y for Ti–6Al–7Nb–0.3Y alloy (Figure 2a,b, Table 3); Ti, Al, Nb, and Ce for Ti–6Al–7Nb–0.3Ce (Figure 2c,d, Table 3); and Ti, Al, Nb, and La for Ti–6Al–7Nb–0.3La alloy (Figure 2e,f, Table 3).

Analysis of REE-rich regions on the alloy’s surfaces indicated the following elemental composition of the selected spots: Titanium, 53.35 ± 0.69 wt. %; Aluminum, 3.32 ± 0.13 wt. %; Niobium, 4.43 ± 0.52 wt. %; and Yttrium, 38.89 ± 0.47 wt. % for Ti–6Al–7Nb–0.3Y (Table 3, Figure 2a,b); Titanium, 49.72 ± 0.56wt. %; Aluminum, 3.80 ± 0.17 wt. %; Niobium, 4.84 ± 0.30 wt. %; and Lanthanum, 41.64 ± 0.57 wt. % for Ti–6Al–7Nb–0.3La (Table 3, Figure 2c,d); Titanium, 82.11 ± 0.74 wt. %; Aluminum, 7.03 ± 0.25wt. %; Niobium, 4.66 ± 0.29wt. %; and Cerium, 3.34 ± 0.33 wt. % for Ti–6Al–7Nb–0.3Ce (Table 3, Figure 2e,f).

The selected areas were analyzed and showed that Ti–6Al–7Nb–0.3Y had the following elemental composition on the selected area—Titanium, 88.69 ± 0.73wt.%; Aluminum, 5.87 ± 0.21 wt.%; Niobium, 5.35 ± 0.29 wt.%; and Yttrium 0.08 ± 0.02 wt.% (Table 3, Figure 2b). Ti–6Al–7Nb–0.3Ce had the following elemental composition—Titanium, 86.70 ± 0.72 wt.%; Aluminum, 7.03 ± 0.25 wt.%; Niobium, 4.66 ± 0.29 wt.%; and Cerium. 1.61 ± 0.22 wt.% (Table 3, Figure 2d). Ti–6Al–7Nb–0.3La had the following elemental composition on the selected area—Titanium, 86.69 ± 0.73 wt.%; Aluminum, 3.13 ± 0.13 wt.%; Niobium, 6.22 ± 0.39 wt.%; and Lanthanum, 3.96 ± 0.31 wt.% (Table 3, Figure 2f). The EDS analysis successfully detected Y, Ce, and La in their respective alloys (Figure 2a,c,e). However, the measured dopant concentrations and their associated standard deviations indicated slight compositional heterogeneity at the microscale. The high standard deviations, particularly for the low-concentration dopants, are attributed to the detection limits of the EDS technique for trace elements and the inhomogeneous distribution of rare-earth elements within the alloy matrix.

**Figure 3:**
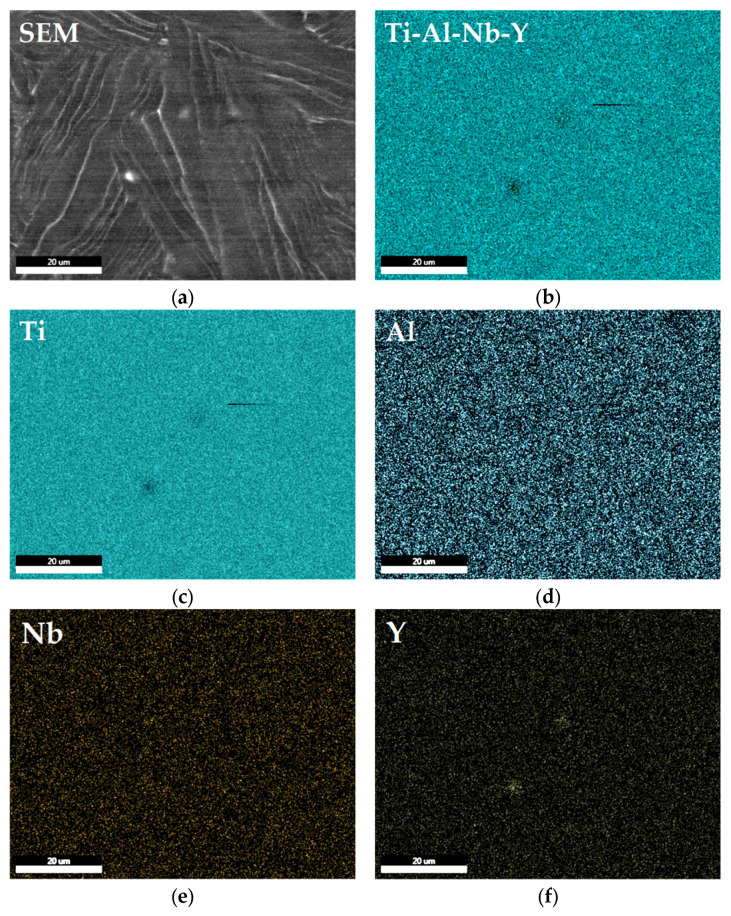
Distribution of elements in the Ti–6Al–7Nb–0.3Y alloy: (**a**) SEM image of the analyzed region, (**b**) overlay map combining Ti (emerald), Al (green), Nb (orange) and Y (yellow), (**c**–**f**) corresponding EDS elemental maps for (**c**) Ti, (**d**) Al, (**e**) Nb, and (**f**) Y.

Element distribution of Ti–6Al–7Nb–0.3Y alloy by means of EDS mapping and SEM analysis showed a quite fine distribution of Ti, Al, Nb, and Y elements within the α–Ti of Ti–6Al–7Nb–0.3Y alloy (Figure 3). It indicated two Yttrium-rich regions with diameter 0.2 μm and 1 μm in the interest location areas related to Y–Al intermetallides (Figure 3d,e). It did not find some non-metallic inclusions such as oxides and nitrides. Also, there were no Y-rich, Nb-rich or Al-rich regions confirmed, nor different Titanium-based phases such as β–Ti phase or AlTi intermetallides along grain boundaries and between grains (Figure 3a,d,e,f).

**Figure 4:**
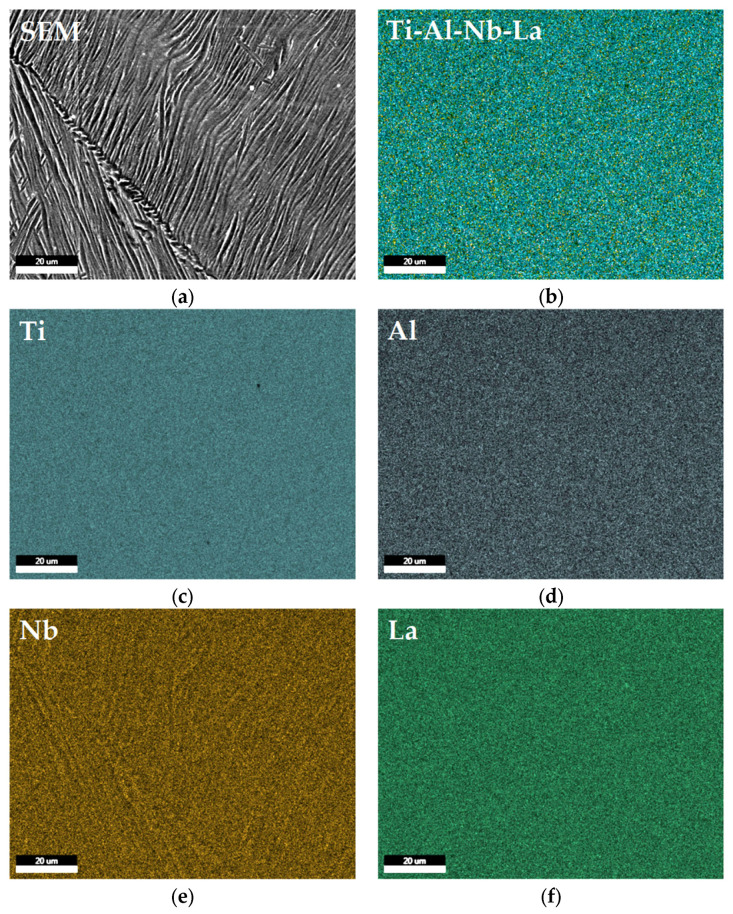
Distribution of elements in the Ti–6Al–7Nb–0.3La alloy: (**a**) SEM image of the analyzed region, (**b**) overlay map combining Ti (emerald), Al (blue), Nb (orange) and La (green), (**c**–**f**) corresponding EDS elemental maps for (**c**) Ti, (**d**) Al, (**e**) Nb, and (**f**) La.

The elemental distribution in the samples of Ti–6Al–7Nb–0.3La alloy was investigated by EDS mapping (Figure 4). EDS elemental mapping confirmed the homogeneous distribution of Ti, Al, Nb and La within the α–Ti and β–Ti matrix phases (Figure 4c–f) with good Lanthanum distribution in the alloy. In contrast, La was not found predominantly segregated at grain boundaries and within interdendritic regions, forming inclusions of La-rich regions or intermetallic particles along grain boundaries and between grains.

**Figure 5:**
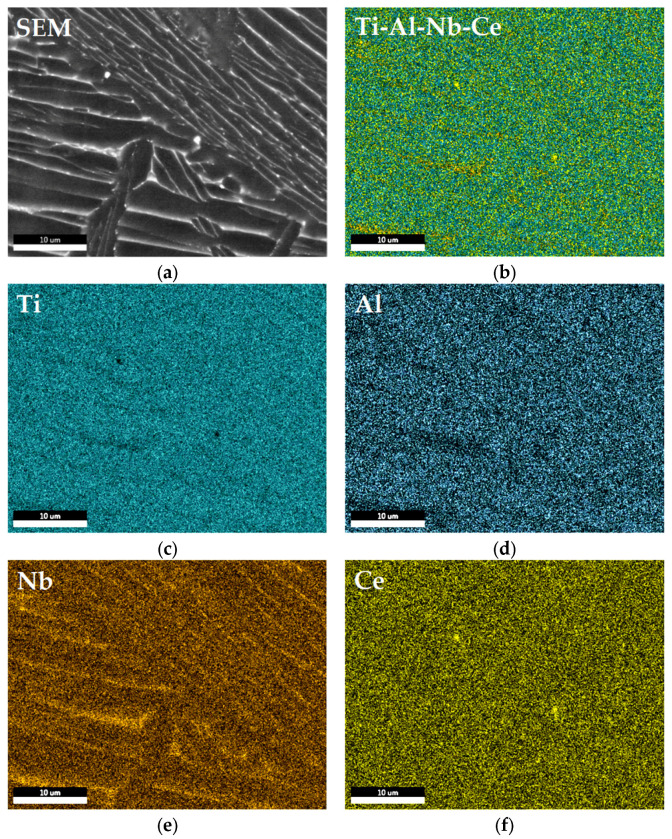
Distribution of elements in the Ti–6Al–7Nb–0.3Ce alloy: (**a**) SEM image of the analyzed region, (**b**) overlay map combining Ti (emerald), Al (green), Nb (orange) and Ce (yellow); (**c**–**f**) corresponding EDS elemental maps for (**c**) Ti, (**d**) Al, (**e**) Nb, and (**f**) Ce.

According to SEM analysis and EDS mapping of Ti–6Al–7Nb–0.3Ce alloy, there were well-distributed compositions of Ti, Al, Nb and Ce elements with Ce-rich and Nb-rich regions (Figure 5). Titanium (Ti) was distributed homogeneously in the sample (Figure 5a). Aluminum (Al) was distributed evenly in the alloy, but it was indicated that there were lower-Aluminum-concentration areas of various isometric shapes and sizes. Also, it indicated Nb-rich regions in the alloy between acicular needle-like microstructures, typical for the hexagonal α–Ti phase (Figure 5c). At the same time, lower Aluminum (Al) content is clearly visible in areas enriched with Niobium (Nb). EDS mapping showed a slight amount of Ce-rich regions’ inclusions, less than 0.5 μm in diameter and finely distributed (Figure 5f). The regions enriched with Cerium are correlated with the Aluminum map; it could be indicated as an Al–Ce intermetallic compound (Figure 5a,c,d).

X-ray elemental analysis of polished alloy samples’ surfaces with diameter 11 mm indicated that Ti–6Al–7Nb–0.3Y alloy comprised 87.61 wt.% of Titanium, 6.23 wt.% of Aluminum, 5.79 wt.% of Niobium, 0.195 wt. % of Vanadium, 0.114 wt. % of Yttrium, and 0.097 wt.% of Cerium (Table 4). Titanium-based alloy with an initial composition of Ti–6Al–7Nb–0.3Ce consisted of 86.6 wt.% of Titanium, 6.72 wt.% of Aluminum, 5.52 wt.% of Niobium, 0.296 wt.% of Iron, 0.215 wt. % of Vanadium, 0.202 wt.% of Chromium, and 0.250 wt.% of Cerium; Ti–6Al–7Nb–0.3La consisted of 85.1 wt. % of Titanium, 7.67 wt. % of Aluminum, 6.06 wt.% of Niobium, 0.34 wt. % of Iron, 0.134 wt.% of Vanadium, 0.23 wt. % of Chromium, and 0.287 wt. % of Lanthanum (Table 4).

A comparative analysis reveals systematic shifts in the Titanium, Aluminum, and Niobium balances across the three Ti–6Al–7Nb–0.3REE (REEs—Y, La, Ce) alloys. The Titanium content shows a gradual decrease from 87.61 wt.% in the Ti–6Al–7Nb–0.3Y alloy to 86.6 wt.% in the Ti–6Al–7Nb–0.3Ce alloy, and further to 85.1 wt.% in the Ti–6Al–7Nb–0.3La alloy. This trend is inversely correlated with the Aluminum (Al) content, which increases progressively from 6.23 wt.% to 7.67 wt.%. Niobium (Nb) content remains relatively stable, ranging from 5.52 to 6.06 wt.% (Table 4).

The Ti–6Al–7Nb–0.3Y alloy demonstrates the highest purity, with Vanadium (0.195 wt.%) as the only detected trace element, aside from Cerium, mentioned above. In contrast, the 0.3 wt. % Ce- and La-microdoped Ti–6Al–7Nb alloys contain measurable quantities of Iron (Fe)—0.296 wt.% and 0.34 wt.%, respectively—Vanadium (V), and Chromium (Cr)—0.202 wt.% and 0.23 wt.%, respectively. These elements are common β–Ti phase stabilizers. Their concurrent presence, particularly Iron (Fe) and Chromium (Cr), could have a synergistic effect on stabilizing the β phase. This could lead to a finer, more refined two-phase (α + β) microstructure upon cooling, which may contribute to the enhanced mechanical strength observed in the Ti–6Al–7Nb–0.3Ce, offsetting the softening tendency expected from its lower Titanium (Ti) content (Table 4).

Reducing melting method showed 0.35 ± 0.02 wt.% of oxygen, 0.14 ± 0.01 wt.% of nitrogen, and 0.011 ± 0.001 wt.% of hydrogen impurities for Ti–6Al–7Nb–0.3Y alloy; 0.18 ± 0.02 wt. % of oxygen, 0.077 ± 0.004 wt. % of nitrogen, and 0.010 ± 0.002 wt. % of hydrogen for Ti–6Al–7Nb–0.3Ce alloy; and 0.25 ± 0.02 wt.% of oxygen, 0.028 ± 0.005 wt. % of nitrogen, and 0.010 ± 0.002 wt. % of hydrogen for Ti–6Al–7Nb–0.3La alloy (Table 5). It should be noted that within this screening study focused on REE effects, the melting protocol was not optimized to guarantee adherence to these interstitial limits for each alloy.

Ti–6Al–7Nb–0.3Y alloy exhibited the highest oxygen concentration at 0.35 ± 0.02 wt.%, a value that approaches the upper limit for many commercial α + β Titanium alloys. In contrast, the Ti–6Al–7Nb–0.3Ce alloy demonstrated the lowest oxygen pickup at 0.18 ± 0.02 wt.%, a reduction of nearly 50% compared to Ti–6Al–7Nb–0.3Y. Ti–6Al–7Nb–0.3La showed an intermediate oxygen level of 0.25 ± 0.02 wt.%. This hierarchy suggests that Cerium may act as a more effective deoxidizer during the melting process compared to Ti–6Al–7Nb–0.3Y or Ti–6Al–7Nb–0.3La alloys, potentially forming stable oxide inclusions that are sequestered from the Titanium matrix.

A parallel trend is observed for nitrogen impurities for Ti–6Al–7Nb–0.3REE (REEs—Y, La, Ce) alloys. Ti–6Al–7Nb–0.3Y contains the highest nitrogen level (0.14 ± 0.01 wt.%), followed by the Ti–6Al–7Nb–0.3Ce (0.077 ± 0.004 wt.%), with the Ti–6Al–7Nb–0.3La possessing the lowest concentration (0.028 ± 0.005 wt.%). The low nitrogen in the Lanthanum-microdoped Ti–6Al–7Nb alloy is particularly noteworthy. All three alloys of Ti–6Al–7Nb–0.3REE (REEs—Y, La, Ce) maintained hydrogen concentrations at a consistently low and safe level (0.010—0.011 wt.%), well below the threshold that could lead to hydrogen embrittlement, indicating that the melting and solidification processes were effectively controlled to minimize hydrogen absorption. Analysis of Ti–6Al–7Nb–0.3REE (REEs—Y, La, Ce) alloys by infrared absorption of CO_2_ and SO_2_ gases indicated a very small amount of impurities. A comparable increase in carbon content was observed from the Ti–6Al–7Nb–0.3Y alloy, 0.019 ± 0.001 wt.%, to the Ti–6Al–7Nb–0.3Ce alloy, 0.023 ± 0.001 wt.%, and finally to the Ti–6Al–7Nb–0.3La alloy, 0.029 ± 0.001 wt.%. The sulfur level was lowest and identical in the Y- and Ce-microdoped Ti–6Al–7Nb alloy, 0.0061 ± 0.0005 wt.%, and was measurably higher in the La-microdoped Ti–6Al–7Nb alloy, 0.0080 ± 0.0005 wt.%.

**Figure 6:**
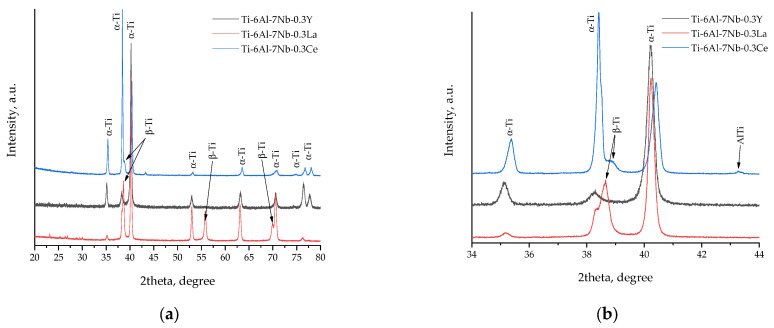
XRD analysis of Ti–6Al–7Nb–0.3REE (REEs—Y, La, Ce) alloys: (**a**) diffractogram area from 20 to 80 degrees of 2 theta indicated α–Ti phase for Ti–6Al–7Nb–0.3Y and both α–Ti and β–Ti phases for Ti–6Al–7Nb–0.3La and Ti–6Al–7Nb–0.3Ce, respectively, (**b**) diffractogram area from 34 to 44 degrees of 2 theta highlighted both α–Ti and β–Ti phases.

In order to confirm phase composition, crystal structure and crystal lattice parameters of Ti–6Al–7Nb–0.3REE (REEs—Y, La, Ce) alloys were studied by XRD analysis (Figure 6).

In alloys Ti–6Al–7Nb–0.3La and Ti–6Al–7Nb–0.3Ce, both α–Ti and β–Ti phases are present, which corresponds to the classical phase composition of the Ti–6Al–7Nb alloy—consisting of the hexagonal α–Ti phase (stabilized by Aluminum) and the regular body-centered β–Ti phase (stabilized by Niobium). However, a significant deviation from this expected composition is observed in the Ti–6Al–7Nb–0.3Ce alloy, characterized by a substantial shift in the α–Ti peaks and a distortion of the crystal lattice (~1.799%). A quantitative analysis of the phase composition reveals that the phase ratios in the Ti–6Al–7Nb–0.3La alloy are as follows: α–Ti to β–Ti = 82 to 18. In the Ti–6Al–7Nb–0.3Ce alloy: α–Ti to β–Ti = 90.5 to 9.5. Furthermore, the Ti–6Al–7Nb–0.3La alloy exhibited an absence of the (200) β–Ti peak, and the (211) phase exhibited an extremely low intensity, a consequence of its minimal presence in the sample (Figure 6).

A slight texture demonstration was observed since the peak intensities of certain planes are subject to change (Table 6). Ti–6Al–7Nb–0.3Ce alloy indicated minor peaks of the Titanium Aluminate phase. It suggested the presence of an impurity phase, which may be an unreacted component of the synthesis. It should be noted that Ti–6Al–7Nb–0.3Y alloy had a single α–Ti phase in comparison with Ti–6Al–7Nb–0.3La and Ti–6Al–7Nb–0.3La (Figure 6, Table 6).

We used an approximation that the crystallites in the Ti–6Al–7Nb–0.3REE (REEs—Y, La, Ce) had spherical shapes to calculate coherent scattering area sizes. These calculations were performed using the Debye–Scherrer and Wulff–Bragg equations. The lattice volumes of the α–Ti phase increased, which indicated doping of the Ti–6Al–7Nb alloy with rare-earth elements (REEs). The lattice parameters of the hexagonal close-packed (hcp) α–Ti phase reveal significant deviations from the reference material, indicative of substantial solute-induced lattice strain. The most significant effect is observed in the Ti–6Al–7Nb–0.3Ce alloy, which exhibits a pronounced contraction of its unit cell volume (ΔV = +1.799%). It indicates a measurable reduction in both a-axis (2.925 Å) and c-axis (4.683 Å) parameters in comparison with the α–Ti standard alloys. This substantial lattice strain is a strong indicator of the incorporation of Cerium into the microstructure. The significant atomic radius mismatch between Ti (≈140 pm) and Ce (≈185 pm) suggests that the observed compressive strain could be primarily caused by Cerium atoms acting as a substitutional solute, potentially leading to solid solution strengthening. However, attributing this distortion solely to a substitutional mechanism is premature without direct local chemical analysis (e.g., TEM/EDS). The contribution of fine-scale intermetallic precipitates, residual stresses, or complex interactions with other alloying elements must also be considered to fully explain the lattice deformation. In contrast, the α–Ti phases in the Ti–6Al–7Nb–0.3Y and Ti–6Al–7Nb–0.3La alloys show a moderate unit cell expansion (ΔV = +0.269% and +0.595%, respectively). The expansion in the Ti–6Al–7Nb–0.3Y alloy can be attributed to the combined effects of Yttrium solubility and its notably higher interstitial oxygen content, 0.35 wt.% (Table 5), as oxygen is a potent α–Ti stabilizer known to expand the crystal lattice. The intermediate expansion in the Ti–6Al–7Nb–0.3La alloy suggests a different balance of solute and interstitial effects (Table 7).

The size of the coherent scattering domains (crystallite size, D) for the α-phase, calculated using the Debye–Scherrer equation, varies notably. The single-phase Ti–6Al–7Nb–0.3Y alloy exhibits the finest α–Ti crystallites (22.32 nm), which is consistent with a microstructure refined by the pinning effect of dissolved Yttrium and/or its oxides. The larger α–Ti crystallites in the dual-phase Ti–6Al–7Nb–0.3Ce and Ti–6Al–7Nb–0.3La (30.77 nm and 29.83 nm, respectively) suggest that the presence of the β phase at elevated temperatures may have provided a different kinetic environment for grain growth during cooling (Table 7). The estimated standard uncertainty for lattice parameters a and c is ±0.005 Å.

The lattice parameters of the body-centered cubic (bcc) β–Ti phase also showed element-specific trends. The β phase in the Ti–6Al–7Nb–0.3Ce alloy displayed a slight contraction (a = 3.279 Å, ΔV = +0.334%), whereas the β phase in the Ti–6Al–7Nb–0.3La alloy showed a more significant expansion (a = 3.290 Å, ΔV = −0.670%) relative to the β–Ti standard alloy. This indicates that while Niobium is the primary β-stabilizer, the specific rare-earth element and associated trace elements (e.g., Fe, Cr) influence the local electronic structure and atomic packing of the metastable β phase. The refined β-phase crystallite sizes (23.34 nm for Ti–6Al–7Nb–0.3Ce, 25.61 nm for Ti–6Al–7Nb–0.3La) further confirm the nanoscale nature of this phase’s distribution (Table 7).

A clear and statistically significant gradient in true density is observed, correlating directly with the atomic mass of the rare-earth element. The measured true densities increase in the following order: Ti−6Al−7Nb−0.3Y (4.4563 ± 0.1075 g/cm^3^) < Ti−6Al−7Nb−0.3Ce (4.7255 ± 0.2853 g/cm^3^) < Ti−6Al−7Nb−0.3La (4.8019 ± 0.0111 g/cm^3^). This progression aligns precisely with the atomic masses of the respective rare-earth metals: Yttrium (88.91 g/mol), Cerium (140.12 g/mol), and Lanthanum (138.91 g/mol). The near-identical masses of Ce and La are reflected in their closely matched densities, while the significantly lighter Y atom results in a measurably lower density for its respective alloy (Table 8).

**Figure 7:**
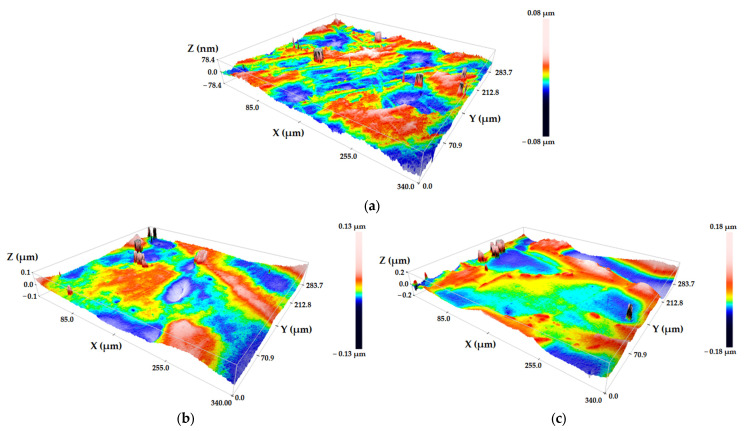
Surface topography images of Ti–6Al–7Nb–0.3REE (REEs—Y, La, Ce) samples before micro- and nanohardness measurements: (**a**) Ti–6Al–7Nb–0.3Y, (**b**) Ti–6Al–7Nb–0.3La, and (**c**) Ti–6Al–7Nb–0.3Ce.

Figure 7 presents quantitative data characterizing the surface topography of Ti–6Al–7Nb–0.3REE (REEs—Y, La, Ce) alloy samples using two standard roughness parameters: the arithmetic mean roughness (Ra) and the maximum height of the profile (Rz).

**Figure 8:**
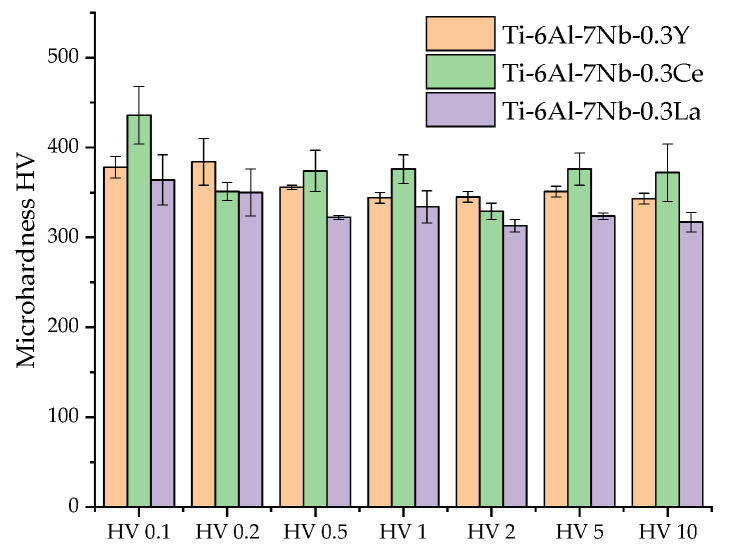
Microindentation results of Ti–6Al–7Nb–0.3REE (REEs—Y, La, Ce) alloys under different loads on indenter—HV 0.1, HV 0.2., HV 0.5, HV 1, HV 2, HV 5, and HV 10.

Microindentation showed different dependencies of Vickers hardness under different loading forces of Ti–6Al–7Nb–0.3REE (REEs—Y, Ce, La). It was indicated that middle hardness for Ti–6Al–7Nb–0.3Y alloy equaled 378 ± 12 HV under 0.1 kgf, 384 ± 26 HV under 0.2 kgf, 355.7 ± 2.3 HV under 0.5 kgf, 344 ± 6 HV under 1 kgf, 345 ± 6 HV under 2 kgf, 351 ± 6 HV under 5 kgf, and 343 ± 6 HV under 10 kgf. Increases in microhardness for Ti–6Al–7Nb–0.3Ce alloy equaled 436 ± 32 HV under 0.1 kgf, 351 ± 10 HV under 0.2 kgf, 374 ± 23 HV under 0.5 kgf, 376 ± 16 HV under 1 kgf, 329 ± 9 HV under 2 kgf, 376 ± 18 HV under 5 kgf, and 372 ± 32 HV under 10 kgf. The lowest microhardness for Ti–6Al–7Nb–0.3La alloy equaled 364 ± 28 (HV 0.1), 350 ± 26 (HV 0.2), 322.3 ± 2.3 (HV 0.5), 334 ± 18 (HV 1), 313 ± 7 (HV 2), 323.7 ± 3.5 (HV 5), and 317 ± 11 (HV 10) (Figure 8, Table 9).

Ti–6Al–7Nb–0.3Y alloy exhibited a characteristic indentation size effect (ISE), where the microhardness decreased markedly from 378 ± 12 HV at 0.1 kgf to a plateau of approximately 344–345 HV at loads above 1 kgf. This ~ 9% reduction in hardness with increasing load is indicative of a material whose resistance to plastic deformation is more significant at shallow indentation depths (Figure 8, Table 9).

Ti–6Al–7Nb–0.3Ce alloy displayed a more complex non-monotonic trend. It registered the peak hardness value of 436 ± 32 HV at 0.1 kgf, which then declined sharply by over 24% to a minimum of 329 ± 9 HV at 2 kgf. Interestingly, the hardness subsequently recovered considerably to 376 ± 18 HV and 372 ± 32 HV at 5 kgf and 10 kgf, respectively. This recovery suggests a potential transition in the dominant deformation mechanism [188,189], possibly involving the interaction of dislocations with Cerium-containing precipitates at higher strain levels (Figure 8, Table 9).

Among the three Ti-based alloys, the Ti–6Al–7Nb–0.3La composition consistently demonstrated the lowest microhardness profile, decreasing gradually from 364 ± 28 HV at 0.1 kgf to 317 ± 11 HV at 10 kgf. This overall reduction of ~13% underscores the comparatively limited strengthening effect of Lanthanum addition across the investigated load range (Figure 8, Table 9).

**Figure 9:**
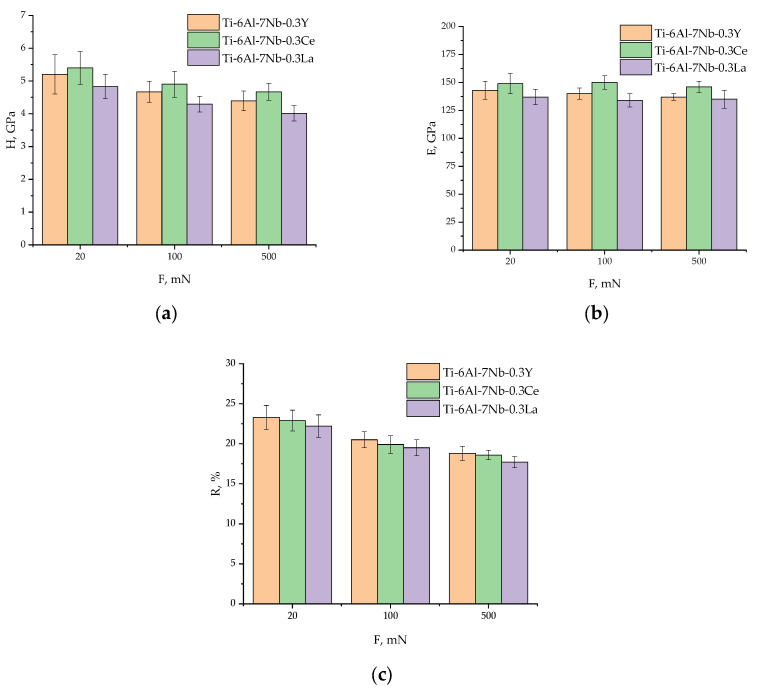
Nanoindentation results of Ti–6Al–7Nb–0.3REE (REEs—Y, La, Ce) alloys under different loads on Berkovich’s indenter—20 mN, 100 mN and 500 mN: (**a**) nanohardness H, (**b**) elastic modulus E, and (**c**) the proportionality of the elastic deformation during indentation R.

Nanoindentation testing using Berkovich’s indenter at loads of 20, 100, and 500 mN was performed to characterize the micromechanical properties of the Ti–6Al–7Nb–0.3REE alloys. The results revealed a clear load dependence and a significant influence on the rare-earth element type (Figure 9, Table 10).

An indentation size effect (ISE) was observed for all alloy compositions of Ti–6Al–7Nb–0.3REE (REEs—Y, La, Ce), with nanohardness decreasing progressively as the applied load increased from 20 to 500 mN (Figure 9, Table 10).

Ti–6Al–7Nb–0.3Ce alloy demonstrated the highest resistance to plastic deformation across the entire load range, with its nanohardness declining from 5.40 ± 0.5 GPa at 20 mN to 4.67 ± 0.26 GPa at 500 mN. In comparison, the Ti–6Al–7Nb–0.3Y alloy exhibited an intermediate nanohardness profile (5.20 ± 0.6 GPa to 4.39 ± 0.30 GPa), while the Ti–6Al–7Nb–0.3La alloy consistently displayed the lowest values, decreasing from 4.83 ± 0.37 GPa to 4.01 ± 0.23 GPa (Figure 9a, Table 10). The elastic modulus, a measure of stiffness, also showed a subtle decreasing trend with increasing load for the Y- and Ce-microdoped alloys (Figure 9b, Table 10). Notably, the Ce-microdoped alloy possessed the highest modulus, peaking at 150 ± 6 GPa at 100 mN, which is approximately 8–12% higher than that of the Ti–6Al–7Nb–0.3La alloy at equivalent loads. This indicates that Ce microdoping provides the most substantial strengthening effect in the elastic modulus (Figure 9b, Table 10). The elastic recovery, representing the proportion of elastically recovered deformation, gradually diminished with increasing indentation depth for all specimens (Figure 9c, Table 10).

The Ti–6Al–7Nb–0.3Y alloy showed a marginally superior elastic recovery, particularly at the highest load of 500 mN (18.8 ± 0.9%), compared to the Ti–6Al–7Nb–0.3Ce alloy (18.6 ± 0.6%) and Ti–6Al–7Nb–0.3La alloys (17.7 ± 0.7%). This suggests a slightly enhanced ability to recover from deformation after the load is removed (Figure 9c, Table 10).

The systemic and local biocompatibility of the experimental Ti–6Al–7Nb–0.3REE (REEs—Y, Ce, La) alloys was evaluated over a 28-day implantation period and compared against a Pure Titanium control. Assessment of general health, body weight, total body temperature, and local body temperature at the implantation site provided a multi-faceted view of the host response.

The general health status of the rats in all groups was satisfactory throughout the experiment. No deaths of rats have been fixed. There were no abnormalities in feed and water intake, nor any infectious and neurological complications. The condition of the skin and hair and the color of the mucous membranes did not change. The behavioral features of the animals and their general conditions corresponded to the expected clinical picture. During rest and movement, the animals assumed a natural physiological position. In all groups, there were no significant changes in body weight at different periods of the experiment compared to the initial values (Table 11).

Body weight served as a key indicator of metabolic and systemic health. As detailed in Table 10, longitudinal monitoring revealed no statistically significant fluctuations in body weight in any group compared to their respective baseline (Day 0) values. While absolute weight differences existed between groups at baseline (e.g., Group 2 and Group 3 were initially heavier), each group maintained a stable trajectory over time. The Control Group (Pure Titanium) and Group 1 (Ti–6Al–7Nb–0.3Y) showed remarkable stability, with median (Me) weights varying by less than 3%. Groups 2 and 3 (La and Ce) also maintained stable weights, with any minor variations falling within the interquartile ranges (Q1–Q3). This sustained weight stability across all cohorts strongly suggests an absence of chronic systemic stress or metabolic disruption attributable to the implant materials.

A significant decrease in the rats’ core body temperature was recorded and post-operatively compared to pre-operative values at all stages in all groups. No significant differences were found between the groups.

As presented in Table 12, a statistically significant decrease (*p* < 0.05) in core body temperature was observed at all post-operative time points (Days 7–28) compared to pre-operative baselines (Day 0). This phenomenon is a well-documented non-specific stress response to surgical trauma and anesthesia. Critically, no statistically significant differences were detected between the experimental groups №1–3 and the Pure Titanium Control Group at any stage. This indicates that the systemic inflammatory and stress response triggered by the implantation of Ti–6Al–7Nb–0.3REE (REEs—Y, Ce, La) alloys was not significantly different in magnitude or duration from the response to the clinically established Pure Titanium control.

Analysis of the dynamics of rats’ local body temperature in the area of implantation of test samples showed that after 7 days of the experiment, in the Control Group and Group 1, there was a significant decrease in this parameter compared to the initial values, by 1.6 °C (*p* = 0.0002; *p* = 0.01, respectively). During this period, a significant increase in local body temperature was recorded in Group 2, by 0.9–1.2 °C, compared to other groups. Subsequently, this parameter in all groups was significantly lower than the initial values, but there were no differences found within each group and between groups. The dynamics of changes in rats’ local body temperature in the area of implantation of Pure Titanium and Ti–6Al–7Nb–0.3REE (REEs—Y, La, Ce) samples is shown in Table 13.

The local body temperature at the implantation site was measured as a direct indicator of the localized inflammatory response. The data, detailed in Table 12, reveals a pattern of the initial tissue–alloy interaction. All groups, including the control, exhibited a significant reduction in local temperature from baseline by Day 14 onwards, consistent with the resolution of the acute inflammatory phase and the progression into tissue repair and remodeling. The most salient finding was observed on Day 7. While the Control (Pure Titanium) and Group 1 (Ti–6Al–7Nb–0.3Y) alloys showed a significant decrease in local temperature, Group 2 (Ti–6Al–7Nb–0.3La) demonstrated a distinct and statistically significant increase in local temperature (Me = 34.2 °C) compared to both its own baseline and all other groups (*p* < 0.05). This transient local hyperthermia is a classic sign of an acute inflammatory response, suggesting that the Ti–6Al–7Nb–0.3La alloy initially provoked a more pronounced local tissue reaction than its counterparts. However, by Day 14, the local temperature for Group 2 had decreased and normalized with the other groups, indicating that this inflammatory response was self–limiting and resolved promptly. In contrast, Group 3 (Ti–6Al–7Nb–0.3Ce) and Group 1 (Ti–6Al–7Nb–0.3Y) displayed a local thermal profile that was largely indistinguishable from Pure Titanium control throughout the study. Ti–6Al–7Nb–0.3Ce, in particular, showed no significant temperature deviation on Day 7, suggesting a minimal initial inflammatory response and a biocompatibility profile comparable to, or even exceeding, that of Pure Titanium. No violations of thermoregulation and energy metabolism processes were detected in the organs of experimental rats in all groups during in vivo experiments, when assessing the safety of new tested materials Ti–6Al–7Nb–0.3 REE (REEs—Y, Ce, La) by means of lifetime research methods (Table 13).

The serum biochemical profile of Wistar rats assessed 3 months post-implantation revealed distinct patterns associated with the different groups. Significant alterations were primarily observed in groups implanted with Ti–6Al–7Nb–0.3La (Group 2) and Ti–6Al–7Nb–0.3Ce (Group 3). Rats in Group 2 exhibited a statistically significant increase in alanine aminotransferase (ALT) activity compared to the control: 102 (94–111) U/L vs. 60 (51–69) U/L, *p* = 0.05. Both Group 2 and Group 3 showed marked elevations in lactate dehydrogenase (LDH) activity—2797 (2335–3352) U/L and 2165 (1990–2569) U/L, respectively—in comparison with the Control Group—1089 (1009–1250) U/L, with *p*-values of 0.05 and 0.04. Similarly, creatinine levels were significantly higher in these two groups, 61 (60–62) µmol/L and 61 (59–61) µmol/L, respectively, than in controls, 54 (52–57) µmol/L, with *p* = 0.05 and *p* = 0.04 (Table 13). The concentration of low- and medium-molecular-weight substances (LMMWSs) was also elevated in Group 2—6.82 (6.61–7.27) conv. units, *p* = 0.05—and Group 3—6.86 (6.76–7.35) conv. units, *p* = 0.04—relative to the Control Group—5.80 (5.43–5.98) conv. units. Catalase activity was significantly increased in Group 1, up to 16.6 (15.6–17.2) %, *p* = 0.05, and Group 3, up to 17.4 (16.0–19.1) %, *p* = 0.05, compared to the Control Group, 14.5 (14.1–15.0) % (Table 14).

No statistically significant differences from the control were found for total protein, C-reactive protein (CRP), aspartate aminotransferase (AST), or urea levels in any experimental group. A notable decrease in blood glucose was observed in Group 2 and Group 3, although these differences did not reach the pre-defined threshold for statistical significance (*p* ≥ 0.05).

Ti–6Al–7Nb–0.3Y alloy had a single-phase hexagonal close-packed (hcp) α–Ti microstructure with fine and acicular needles. It showed the finest α–Ti crystallite size (22.32 nm). Both Ti–6Al–7Nb–0.3La and Ti–6Al–7Nb–0.3Ce alloys formed a dual phase α + β microstructure. A notable 1.8% lattice contraction of α–Ti in Ti–6Al–7Nb–0.3Ce alloy indicated substantial substitutional solid solution strengthening due to the atomic size mismatch between Ti and Ce. EDS mapping confirmed a generally homogeneous distribution of alloying elements. Local Y-rich regions (0.2–1 μm) in Ti–6Al–7Nb–0.3Y and Ce-rich regions (<0.5 μm) in Ti–6Al–7Nb–0.3Ce were observed, suggesting the formation of minor intermetallic phases or clusters in microscale. The Ti–6Al–7Nb–0.3Ce alloy demonstrated the most effective deoxidizing capability, with the lowest oxygen content (0.18 wt.%). The Ti–6Al–7Nb–0.3Y alloy had the highest oxygen (0.35 wt.%) and nitrogen (0.14 wt.%) levels. All alloys had low hydrogen levels. The Ti–6Al–7Nb–0.3La and Ti–6Al–7Nb–0.3Ce alloys contained measurable amounts of β-stabilizing impurities (Fe, Cr, V), which were absent in Ti–6Al–7Nb–0.3Y alloy. This is postulated to synergistically affect β-phase stability. Ti–6Al–7Nb–0.3Ce alloy exhibited the highest peak hardness (436 HV at 0.1 kgf) and a complex, non-monotonic load dependence, indicating potent strengthening mechanisms. Ti–6Al–7Nb–0.3La alloy consistently showed the lowest hardness values. Nanoindentation confirmed the superior mechanical performance of the Ti–6Al–7Nb–0.3Ce alloy, which possessed the highest nanohardness (up to 5.40 GPa) and elastic modulus (up to 150 GPa) across all loads. All alloys exhibited an indentation size effect (ISE). All implanted groups, including the Pure Titanium control, showed a similar, transient post-operative decrease in core body temperature—a normal stress response. No significant differences in body weight or general health were observed. On Day 7, the Ti–6Al–7Nb-0.3La (Group 2) alloy induced a significant local temperature increase, indicating a more pronounced acute inflammatory reaction compared to other groups. This response was resolved by Day 14. Serum analysis revealed significant hepatotoxic and nephrotoxic effects for Ti–6Al–7Nb–0.3La (Group 2) and Ti–6Al–7Nb–0.3Ce (Group 3) alloys, evidenced by elevated ALT, LDH, creatinine, and low-molecular-weight substances. The Ti–6Al–7Nb–0.3Y (Group 1) alloy showed a compensated response (elevated catalase activity) without significant signs of organ damage, presenting a biocompatibility profile closest to Pure Titanium.

## 4. Discussion

The near-alpha Ti–6Al–7Nb alloy typically has a microstructure dominated by the HCP α–Ti phase, with a minor fraction of the BCC β–Ti phase, stabilized primarily by Niobium. The addition of 0.3 wt.% Y, La, and Ce altered the classic α + β morphology, leading to a refinement of α–Ti phase colonies and a more discontinuous distribution of β–Ti phase at the α–Ti grain boundaries (Figure 1). This suggests that the REEs acted as grain refinement agents in the case of the Ti–6Al–7Nb–0.3Y alloy (Figure 1a,b and Figure 3a,b) and influenced the β–Ti phase stability in the case of the Ti–6Al–7Nb–0.3La alloy (Figure 1c,d and Figure 5a,b) and Ti–6Al–7Nb–0.3Ce (Figure 2e,f and Figure 6a,b).

The measured compositions align well with the target Ti–6Al–7Nb–0.3REE (REEs—Y, La, Ce) stoichiometry. The REEs were successfully integrated into the matrix, with measured concentrations of 0.114 wt.% of Yttrium, 0.250 wt.% of Cerium, and 0.287 wt.% of Lanthanum (Table 4). The slight deviations from the nominal 0.3 wt.% can be attributed to standard manufacturing losses, such as oxidation or evaporation during the melting process. It is noteworthy that the Ti–6Al–7Nb–0.3Y alloy exhibited a trace amount of Cerium (0.097 wt.%), suggesting a minor impurity in the starting Yttrium feedstock or cross-contamination during processing (Table 4). This incidental inclusion, while small, could potentially influence the alloy’s microstructural evolution.

A comparative analysis reveals systematic shifts in the Titanium, Aluminum, and Niobium balances across the three Ti–6Al–7Nb–0.3REE (REEs—Y, La, Ce) alloys. The Titanium content shows a gradual decrease from 87.61 wt.% in the Ti–6Al–7Nb–0.3Y alloy to 86.6 wt.% in Ti–6Al–7Nb–0.3Ce alloy, and further to 85.1 wt.% in the Ti–6Al–7Nb–0.3La alloy. This trend is inversely correlated with the Aluminum content, which increases progressively from 6.23 to 7.67 wt.%. Niobium content remains relatively stable, ranging from 5.52 to 6.06 wt.% (Table 4).

These variations are significant as they directly impact the phase stability and microstructural constituents of the Ti–6Al–7Nb–0.3REE (REEs—Y, La, Ce) alloys. The Al content acts as a potent α–Ti phase stabilizer, while Nb is a β–Ti phase stabilizer. The higher Al content in the Ti–6Al–7Nb–0.3La alloy suggests a potentially larger volume fraction of the α–Ti phase at room temperature compared to the Ti–6Al–7Nb–0.3Y and Ti–6Al–7Nb–0.3Ce alloys (Figure 6, Table 6). This compositional shift could be a primary factor underlying the differences in mechanical properties, such as the lower hardness, 4.01 GPa under a load of 500 mN, observed in Ti–6Al–7Nb–0.3La alloy (Table 10).

For all three alloys of Ti–6Al–7Nb–0.3REE (REEs—Y, La, Ce), hardness decreases monotonically as the load increases from 20 mN to 500 mN. This is physically attributed to the greater influence of geometrically necessary dislocations (GNDs) at shallow indentation depths. At lower loads (e.g., 20 mN), the high strain gradient within a small, confined plastic zone necessitates a high density of GNDs, resulting in elevated hardness values. As the load and indentation depth increase, the role of GNDs diminishes relative to the statistically stored dislocation population, leading to a decrease in the measured hardness (Figure 9, Table 10). All these effects were studied and confirmed by Fu, Y. et.al., Wang Q. et.al., Nath P. et.al., Demir E. et.al., Widjaja A. et.al., and Voyiadjis G.Z. et.al. of [190,191,192,193,194,195]. However, the Ce-containing alloy demonstrates a clear deviation from this pattern, characterized by a non-monotonic trend and significant scatter. This behavior is attributed to pronounced microstructural heterogeneity, where the indenter probes a dual-phase matrix alongside fine intermetallic precipitates. Therefore, while nanoindentation effectively characterizes local resistance to deformation, the observed anomaly for the Ti–6Al–7Nb–0.3Ce alloy explicitly indicates that its overall mechanical performance—especially critical for implant applications—must be assessed via bulk-scale tensile and fatigue testing.

Table 10 shows that Ti–6Al–7Nb–0.3Ce alloy exhibits superior resistance to plastic deformation across the entire load spectrum. It possesses the highest hardness at 20 mN (5.40 ± 0.5 GPa) and maintains this advantage at 500 mN (4.67 ± 0.26 GPa); on the other hand, Ti–6Al–7Nb–0.3Y shows an intermediate profile, while Ti–6Al–7Nb–0.3La consistently demonstrates the lowest hardness values, with a minimum of 4.01 ± 0.23 GPa at 500 mN. This hierarchy suggests that Cerium acts as the most potent strengthener, likely through the formation of fine, stable dispersoids that effectively impede dislocation motion, which was confirmed by earlier research [196]. A subtle yet consistent decrease in elastic modulus is observed for the Ti–6Al–7Nb–0.3Y and Ti–6Al–7Nb–0.3Ce as the load increases. For instance, the modulus of the Ti–6Al–7Nb–0.3Ce decreases from 149 ± 9 GPa at 20 mN to 146 ± 5 GPa at 500 mN. This minor reduction may be attributed to the decreasing influence of surface oxide layers or near-surface residual stress at higher penetration depths. The Ti–6Al–7Nb–0.3La elastic modulus remains relatively constant within error margins. Critically, the Ti–6Al–7Nb–0.3Ce alloy displays the highest elastic modulus at all loads, with a peak value of 150 ± 6 GPa at 100 mN. This represents a significant enhancement of approximately 9% and 12% over the Ti–6Al–7Nb–0.3Y and Ti–6Al–7Nb–0.3La alloys, respectively, at equivalent loads. It indicates that Cerium microdoping most effectively increases the interatomic bonding forces and the overall stiffness of the Titanium matrix, potentially via solid solution strengthening, as indicated by Poorganji B. et.al., Pilchak A.L. et.al.,Xu Y. et.al., Li K.M. et.al., Wheeler D.W. et.al., Chen Y.-H. et.al., Yang Y.F. et.al., Cui J. et.al., Wang Q. et.al.; Zhu Y. et.al., Zhu Y. et.al., Wang X. et.al. of [79,147,148,196,197,198,199,200,201,202,203,204,205].

Under a constant applied load, a shallower indentation depth correlates directly with higher resistance to penetration (hardness). Consistent with its superior hardness, the Ti–6Al–7Nb–0.3Ce consistently exhibits the smallest indentation depths at all loads (e.g., 2266 ± 60 nm at 500 mN). Conversely, the softest alloy, Ti–6Al–7Nb–0.3La, consistently displays the greatest penetration (e.g., 2429 ± 64 nm at 500 mN) (Table 10).

The elastic recovery, which quantifies the fraction of deformation recovered upon unloading, decreases progressively with increasing load for all compositions of Ti–6Al–7Nb–0.3REE (REEs—Y, La, Ce). This trend is expected, as larger plastic zones generated under higher loads lead to a greater proportion of irreversible deformation. Notably, Ti–6Al–7Nb–0.3Y demonstrates a marginally superior elastic recovery at the highest load (18.8 ± 0.9% at 500 mN) compared to the Ti–6Al–7Nb–0.3Ce alloy (18.6 ± 0.6%) and Ti–6Al–7Nb–0.3La alloy (17.7 ± 0.7%) variants. This suggests that the Ti–6Al–7Nb–0.3Y alloy possesses a slightly more resilient microstructure, capable of recovering a greater proportion of its deformation after the indentation load is removed—a property potentially linked to improved resistance to contact damage (Table 10).

Thus, the indentation size effect (ISE) is a dominant factor, with hardness being highly sensitive to the test load [190,191,192,193,198,205,206]. Ti–6Al–7Nb–0.3Ce is the most effective rare-earth element addition for enhancing overall micromechanical performance, providing the highest combination of nanohardness (H), 4.67 GPa under a load of 500 mN, and elastic modulus (E), 146 GPa (Figure 9, Table 10). The Ti–6Al–7Nb–0.3Y alloy offers a balanced profile, with intermediate nanohardness (H), 4.39 GPa under a load of 500 mN, but with a marginally superior ability for elastic recovery, 18.8% at 500 mN (Figure 9, Table 10). Ti–6Al–7Nb–0.3La provides the most modest strengthening effect, resulting in the lowest nanohardness (H), 4.01 GPa, of the three Ti–6Al–7Nb–0.3REE (REE–Y, Ce, La) alloys studied (Figure 9, Table 10).

Mattern N. et.al. of [207] determined that the maximum solid solubility of Lanthanum in Titanium was below 1.0 at. %, specifically finding the La content in the body-centered cubic β–Ti (La) phase at T = 1800 K to be (0.6 ± 0.3) at.%, which corresponds to XRD data. Another study [208] showed good Yttrium solubility (2.33 wt. % or 1.0 at. %) in the Ti–46Al–2Cr–2Nb alloy with α–Ti phase and γ–Ti phase formation, with a slight amount of Al_2_Y phase that was confirmed by XRD. Weng W. et.al. of [209] reported that 0.1 wt. % Yttrium microalloying of Ti–24Nb–38Zr–2Mo alloy resulted in lattice parameters increasing until 3.369 ± 0.003 A, in comparison with 3.369 ± 0.003 A for the basic alloy composition of Ti–24Nb–38Zr–2Mo. For the current research, the XRD patterns show a clear pattern in the phase composition among the three alloys of Ti–6Al–7Nb–0.3REE (Figure 6, Table 6). The Ti–6Al–7Nb–0.3Y alloy exhibits a single-phase hexagonal close-packed (hcp) α–Ti structure, with no detectable peaks corresponding to the body-centered cubic (bcc) β–Ti phase. In contrast, both the Ti–6Al–7Nb–0.3Ce and Ti–6Al–7Nb–0.3La alloys display a classical dual-phase (α + β) microstructure typical of Ti–6Al–7Nb [210]. However, quantitative phase analysis reveals significant differences between these two dual-phase (α + β) alloys. The volume fraction of the α phase is substantially higher for the Ti–6Al–7Nb–0.3Ce alloy (90.5%) compared to the Ti–6Al–7Nb–0.3La alloy (82%), with a corresponding β-phase fraction of 9.5% and 18%, respectively. This indicates that Cerium has a stronger α-stabilizing effect or suppresses the β-phase formation more effectively than Lanthanum under the given processing conditions. Furthermore, the absence of the (200) β–Ti peak and the extremely low intensity of the (211) β–Ti peak at the Ti–6Al–7Nb–0.3La alloy diffractogram suggest a potential texture or a very low volume fraction of the β phase that is near the detection limit of XRD (Figure 6, Table 6 and Table 7).

A detailed analysis of the lattice parameters provides insight into the dissolution and interaction of alloying elements within the Titanium matrix. A critical finding is the significant lattice distortion observed in the α–Ti phase of the Ti–6Al–7Nb–0.3Ce alloy, which exhibits a contraction in unit cell volume (ΔV = +1.799%) relative to the standard α–Ti ICDD reference (01–089–3073). This substantial volumetric contraction is a strong indicator of Cerium atoms entering the Titanium lattice as a substitutional solute, likely inducing localized strain fields due to the atomic size mismatch (Figure 6, Table 7).

The α–Ti phase lattice in the Ti–6Al–7Nb–0.3Y and Ti–6Al–7Nb–0.3La alloys also showed volumetric changes (ΔV = +0.269% and +0.595%, respectively), though less pronounced than in the Ti–6Al–7Nb–0.3Ce alloy (Table 7). The expansion in the Ti–6Al–7Nb–0.3Y is consistent with the known solid solution behavior of Yttrium and the potential influence of its higher oxygen content 0.35 wt. % (Table 5), as oxygen is a potential α–stabilizer that can also expand the lattice [17,211,212]. The presence of minor peaks corresponding to a Titanium Aluminate phase in the Ti–6Al–7Nb–0.3Ce alloy suggests that not all Cerium remained in solid solution, with a fraction likely participating in the formation of secondary intermetallic compounds (Figure 6).

The size of the coherent scattering domains (crystallite size, D), calculated using the Debye–Scherrer equation, varied notably between the alloys. The α–Ti crystallites in the single-phase Ti–6Al–7Nb–0.3Y alloy were the finest (22.32 nm), while the dual-phase Ti–6Al–7Nb–0.3Ce and Ti–6Al–7Nb–0.3La alloys exhibited larger α–Ti phase crystallites (30.77 nm and 29.83 nm, respectively). This suggests that the presence of the β–Ti phase at the processing temperature may have provided a less constrained environment for α–Ti phase grain growth during cooling. The β–Ti phase crystallites in the Ti–6Al–7Nb–0.3Ce and Ti–6Al–7Nb–0.3La were also refined, with sizes of 23.34 nm and 25.61 nm, respectively (Table 7).

Thus, XRD analysis provides a fundamental microstructural explanation for the previously observed mechanical properties: the single-phase α–Ti structure of the Ti–6Al–7Nb–0.3Y alloy, combined with its high interstitial (O, N) content, is a primary contributor to its medium hardness, 4.39 under a load of 500 mN, as both factors provide strong solid solution strengthening (Figure 9, Table 10). However, the presence of the tougher β–Ti phase may be detrimental to its ductility; the high α–Ti phase fraction and significant lattice strain in the Ti–6Al–7Nb–0.3Ce alloy (Figure 6, Table 6), likely due to Cerium in solid solution and fine–scale precipitates (e.g., Titanium Aluminides), are consistent with its superior combination of high hardness, 4.67 ± 0.26 GPa at 500 mN, and high elastic modulus, 146 ± 7 GPa at 500 mN (Figure 9, Table 10). The fine, dispersed β–Ti phase and potential precipitate strengthening offer a balanced strengthening mechanism; the Ti–6Al–7Nb–0.3La alloy, with a more balanced α/β ratio and the least lattice distortion, exhibited the lowest hardness, 4.01 GPa under a load of 500 mN, indicating that Lanthanum provides the most modest strengthening effect among the three rare-earth elements (REEs)—Y, La, and Ce (Figure 9, Table 10).

Other researchers confirmed that in vivo biocompatibility tests of biodegradable alloys of Mg–REE (REEs—Ce, La, Nd), with compositions Mg–1.27Ce, Mg–0.69La, and Mg–2.13Nd, did not have significantly harmful tissue effects. There were no abnormal behaviors or changes in weight, and all three types of Mg–REE implants were slow-degrading and showed good biocompatibility [15]. It was confirmed that Ti–24Nb–38Zr–2Mo–0.1Y exhibited good cytocompatibility on seeding with SaOS2 cells by Weng W. et.al. of [209]. The integrated analysis of these in vivo parameters allows for a comparative assessment of the alloys’ biosafety profiles: all tested Ti–6Al–7Nb–0.3REE (REEs—Y, Ce, La) alloys demonstrated systemic biocompatibility, with no evidence of toxicity, as reflected in stable body weight, normal physiological parameters, and a systemic stress response indistinguishable from the Pure Titanium Control sample (Table 11, Table 12 and Table 13). Changes in general temperature (most often decreased) after surgery reflect the systemic stress response of the body to surgical trauma and anesthesia, which is noted in many works. Body weight is monitored as a standard parameter of animal welfare. Stable body weight of an animal in a long-term experiment is considered an indicator of the absence of systemic toxicity in many in vivo biocompatibility studies [213,214,215]. Table 13 shows that the local tissue response delineates a clear biocompatibility hierarchy: Ti–6Al–7Nb–0.3Ce (Group 3) and Ti–6Al–7Nb–0.3Y (Group 1) exhibited the most favorable profiles, with local inflammatory responses that were minimal and/or equivalent to the Pure Titanium (Control Group). Ti–6Al–7Nb–0.3La (Group 2) induced a transient, but statistically significant, increase in local inflammation at the one-week mark. While this response resolved by the second week, it indicates a comparatively more reactive interface in the acute phase post-implantation (Table 11, Table 12 and Table 13).

Thus, all three Ti–6Al–7Nb–0.3REE alloys (REEs—Y, La, Ce) have passed preliminary biocompatible in vivo tests in comparison to Pure Titanium (Control Group), but the Ti–6Al–7Nb–0.3Y alloy (Group 1) is much more preferable for further research due to minimal and/or equivalent local inflammatory responses, proven by other researchers [15,209,216,217,218]. As for the Ti–6Al–7Nb–0.3La alloy (Group 2) and the Ti–6Al–7Nb–0.3Ce alloy (Group 3), they had statistically significant increases in local inflammation at the one-week mark, which needs further research and explanation as well, as this can be an indicator of toxicity (Table 13), but other researchers did not observe the same effect with the highest concentration of Lanthanum in different alloys for biomedical applications [15,216,219]. An increase in temperature in the implantation area is a direct indicator of inflammation or infection. A study [220] on a rabbit model clearly showed that the local temperature around an infected spinal implant significantly increases.

Current serum data of Wistar rats (Table 14) can be integrated into a comprehensive toxicological profile through a panel of serum biomarkers—encompassing specific organ enzymes (ALT, AST) and inflammatory mediators (CRP)—transcending the role of isolated indicators. It forms an integrated diagnostic signature. In the Wistar rat model, concordant elevations in this signature provide compelling evidence for a pathophysiological sequence: implant biocorrosion → systemic ion distribution → hepatic (and other organ) bioaccumulation → oxidative and inflammatory organ damage → release of damage markers into serum. This mechanistic cascade underscores the high predictive value of serum biochemistry for detecting and monitoring the subtler, systemic toxicological impacts of biomaterials that may not be immediately apparent through histology alone [221,222,223,224,225,226,227,228,229].

The longitudinal analysis of serum biochemical parameters at 3 months post-implantation reveals a complex, multi-organ pathophysiological response to the implanted alloys, characterized by distinct toxicity profiles that correlate with specific rare-earth element doping (Table 14). Elevations in the activities of liver-specific enzymes in the serum, particularly alanine aminotransferase (ALT) and aspartate aminotransferase (AST), are classical hallmarks of hepatocyte damage and compromised membrane integrity [221,222,224,225,226,227,228,230,231,232,233]. The release of these cytosolic enzymes into circulation is a direct consequence of cellular injury, allowing serum measurements to function as a non-invasive, real-time readout of hepatic status. In models assessing the biocompatibility of metallic implants, increased serum ALT and AST levels have been consistently linked to the hepatic bioaccumulation of metal ions, such as Titanium and Aluminum, released via biocorrosion of Titanium [234,235,236]. These ions can disrupt mitochondrial function, induce peroxidative damage to lipid membranes, and trigger apoptotic pathways within hepatocytes, with the resulting cytolysis being faithfully reflected in the serum enzyme profile [25,223,226,233,237,238,239,240,241,242,243,244].

The concurrent elevation of CRP and creatinine observed in this study indicates a state of systemic inflammation coupled with incipient renal stress. This clinical association is well-established, where elevated CRP is recognized as an independent predictor of declining renal function and progressive kidney disease [245,246]. Furthermore, the concomitant alteration in catalase activity alongside elevated CRP reflects the known pathogenic cycle, where inflammatory cytokines stimulate reactive oxygen species production and vice versa [247], a dynamic evidenced by the inverse correlation between CRP levels and antioxidant capacity in chronic inflammatory states [248]. In the context of our Ti–Al–Nb implant mode system, this integrated biomarker signature supports a pathophysiological sequence initiated by biocorrosion and systemic ion release [249], leading to widespread inflammatory and oxidative stress responses that ultimately manifest as subclinical organ stress—a phenomenon directly demonstrated by elevated CRP and hepatorenal markers in animals with Titanium alloy implants. This cascade is detectable through sensitive serum biochemistry before overt histological damage may be apparent.

Thus, long-term systemic toxicity by serum analysis at 3 months post-implantation revealed significant late-stage toxicological effects associated with specific REE (Y, La, Ce) microdopants. Implantation of the Ti−6Al−7Nb−0.3La (Group 2) and Ti−6Al−7Nb−0.3Ce (Group 3) alloys led to sustained hepatotoxic and nephrotoxic effects, evidenced by statistically significant elevations in alanine aminotransferase (ALT) and lactate dehydrogenase (LDH), markers of hepatocellular injury, most pronounced in the Ti−6Al−7Nb−0.3La (Group 2); creatinine, a marker of impaired renal function; and low- and medium-molecular-weight substances (LMMWSs)—indicative of altered metabolic clearance. The Ti−6Al−7Nb−0.3Y alloy (Group 1) did not induce significant elevations in these toxicity markers. It showed a significant increase only in catalase activity, an antioxidant enzyme, suggesting a compensated oxidative stress response without consequent visceral organ damage, and it can be related to a slight Cerium impurity of 0.097 wt. % in the alloy (Table 14).

## 5. Conclusions

This study considers Ti–6Al–7Nb–0.3REE (REEs—Y, Ce, La) novel alloys and sheds light on the effect of ≤0.3 wt. % REE microdoping on microstructure, mechanical properties, and phase composition, as well as studying novel alloys’ biocompatibility by means of an in vivo subcutaneous implantation model on Wistar rats because of previously controversial scientific results about REE doping of alloys and their biocompatibility. We state the following significant conclusions:

1. The addition of Yttrium resulted in a single-phase α-Ti microstructure with the finest crystallites (22.32 nm), attributed to a grain-boundary pinning effect. In contrast, Cerium and Lanthanum promoted the formation of dual-phase α + β structures with coarser α grains (30.77 nm and 29.83 nm, respectively).
2. A clear hierarchy in mechanical performance was established. The Ce-modified alloy exhibited the highest nanohardness (4.67 GPa) and elastic modulus (146 GPa). The Y-containing alloy offered a balanced profile with intermediate hardness (4.39 GPa) and superior elastic recovery (18.8%), while the La-doped alloy showed the most modest strengthening (hardness of 4.01 GPa).
3. XRD analysis linked these properties to underlying structural features. The high hardness of the Y-alloy is explained by interstitial solid solution strengthening (0.35 wt.% O, 0.14 wt.% N) within its single-phase structure. The superior properties of the Ce-alloy correlate with significant lattice strain (ΔV = +1.799%) and a high α-phase fraction, likely due to solute Ce and fine-scale precipitates.
4. The measured true density increased with the atomic mass of the REE: 4.4563 g/cm^3^ (Y) < 4.7255 g/cm^3^ (Ce) < 4.8019 g/cm^3^ (La). In vivo assessment revealed an element-specific biological response. The La-alloy induced a significant but transient local inflammatory reaction (34.2 °C at Day 7). After three months, both La- and Ce-alloys showed signs of systemic hepatotoxicity and nephrotoxicity, evidenced by elevated serum markers (ALT, LDH, creatinine). Crucially, the Y-modified alloy showed a biocompatibility profile statistically indistinguishable from the Pure Titanium control.

This work has identified clear trends, but its interpretation must consider key limitations: (i) uncontrolled variation in interstitial element content between alloys, (ii) the preliminary nature of the in vivo screening model, and (iii) mechanical evaluation limited to indentation techniques. Consequently, the Ti−6Al−7Nb−0.3Y alloy emerges as the most promising candidate based on its refined microstructure, balanced properties, and favorable biocompatibility. Future studies with strict compositional control, comprehensive mechanical testing, and advanced biological characterization are essential to validating these findings and assessing the potential of REE-microdoped Ti−6Al−7Nb for biomedical applications.

## Figures and Tables

**Table 1:** Concentration of rare-earth elements (REEs) causing death of 50% of test organisms [90].

Rare-earth Element	Toxicity LC50 (mg/L)
Yttrium (Y)	27.6
Lanthanum (La)	62.7
Cerium (Ce)	25.6
Neodymium (Nd)	47.6
Gadolinium (Ga)	58.2
Terbium (Tb)	11.4

**Table 2:** Chemical requirements for Ti–6Al–7Nb alloy according to ASTM F1295–24 and ISO 5832–11:2024 standards.

Element	Compositional Limits, wt. % (ASTM F1295–24)	Compositional Limits, wt. % (ISO 5832–11:2024)
Titanium (Ti)	Balance	Balance
Aluminum (Al)	5.50 to 6.50	5.50 to 6.50
Niobium (Nb)	6.50 to 7.50	6.50 to 7.50
Iron (Fe)	0.25 max	0.25 max
Oxygen (O)	0.20 max	0.20 max
Carbon (C)	0.08 max	0.08 max
Nitrogen (N)	0.05 max	0.05 max
Hydrogen (H)	0.009 max	0.009 max
Cobalt (Co)	<0.10	Not specified
Tantalum (Ta)	Not specified	0.50 max
Other Elements, Each	0.10 max	0.10 max
Other Elements, Total	0.40 max	0.30 max
Specific REEs’ Limits	Not specified	Not specified

**Table 3:** EDS elemental analysis of Ti–6Al–7Nb–0.3REE (REEs—Y, La, Ce) alloys in different spots and areas of interest on the samples’ surfaces.

Alloy Composition and EDS Analysis Area	Elements, wt. %			
	Ti	Al	Nb	REE (Y, La, Ce)
Ti–6Al–7Nb–0.3Y, spot	53.35 ± 0.69	3.32 ± 0.13	4.43 ± 0.52	38.89 ± 0.47
Ti–6Al–7Nb–0.3Y, area	88.69 ± 0.73	5.87 ± 0.21	5.35 ± 0.29	0.08 ± 0.02
Ti–6Al–7Nb–0.3La, spot	49.72 ± 0.56	3.80 ± 0.17	4.84 ± 0.30	41.64 ± 0.57
Ti–6Al–7Nb–0.3La, area	86.69 ± 0.73	3.13 ± 0.13	6.22 ± 0.39	3.96 ± 0.31
Ti–6Al–7Nb–0.3Ce, spot	82.11 ± 0.74	5.43 ± 0.20	9.12 ± 0.38	3.34 ± 0.33
Ti–6Al–7Nb–0.3Ce, area	86.70 ± 0.72	7.03 ± 0.25	4.66 ± 0.29	1.61 ± 0.22

**Table 4:** X-ray elemental analysis of Ti–6Al–7Nb–0.3REE (REEs—Y, La, Ce) alloys.

Element, wt. %	Alloy Composition		
	Ti–6Al–7Nb–0.3Y	Ti–6Al–7Nb–0.3Ce	Ti–6Al–7Nb–0.3La
Titanium (Ti)	87.61	86.6	85.1
Aluminum (Al)	6.23	6.72	7.67
Niobium (Nb)	5.79	5.52	6.06
Iron (Fe)	–	0.296	0.34
Vanadium (V)	0.195	0.215	0.134
Chromium (Cr)	–	0.202	0.23
Cerium (Ce)	0.097	0.250	–
Yttrium (Y)	0.114	–	–
Lanthanum (La)	–	–	0.287

**Table 5:** Ti–6Al–7Nb–0.3REE (REEs—Y, La, Ce) alloys’ analysis of oxygen (O), nitrogen (N), and hydrogen (H) by reduction melting and analysis of sulfur (S) and carbon (C) contents by oxidative melting method.

Alloy Composition, wt. %		Oxygen, wt. %	Nitrogen, wt. %	Hydrogen, wt. %	Carbon, wt. %	Sulphur, wt. %
Ti–6Al–7Nb–0.3Y	Average	0.35	0.14	0.011	0.019	0.0061
	S_d_	0.02	0.01	0.001	0.001	0.0005
Ti–6Al–7Nb–0.3La	Average	0.25	0.028	0.010	0.029	0.0080
	S_d_	0.02	0.005	0.002	0.001	0.0005
Ti–6Al–7Nb–0.3Ce	Average	0.18	0.077	0.010	0.023	0.0061
	S_d_	0.02	0.004	0.002	0.001	0.0005

**Table 6:** Ti–6Al–7Nb–0.3REE (REEs—Y, La, Ce) intensity of the XRD peaks of certain planes.

Crystal Orientation	Alloy Composition		
	Ti–6Al–7Nb–0.3Y	Ti–6Al–7Nb–0.3Ce	Ti–6Al–7Nb–0.3La
α—Ti phase			
100	+	+	+
002	+	+	+
101	+	+	+
102	+	+	+
110	+	+	+
103	+	+	+
200	+	+	+
112	+	+	+
201	+	+	+
β—Ti phase			
110	−	+	+
200	−	+	+
211	−	+	+

**Table 7:** Crystal lattice parameters of Ti–6Al–7Nb–0.3REE (REEs—Y, La, Ce) alloys and ICSD data for α–Ti 01–089–3073 and β–Ti 01–089–4913.

Phase Name	a, A	c, A	D, nm	V, A^3^	∆V, %
α–Ti–6Al–7Nb–0.3Y	2.943	4.697	22.32	183.798	0.269
α–Ti–6Al–7Nb–0.3Ce	2.925	4.683	30.77	180.290	1.799
α–Ti–6Al–7Nb–0.3La	2.941	4.688	29.83	182.502	0.595
β–Ti–6Al–7Nb–0.3Ce	3.279	–	23.34	35.266	0.334
β–Ti–6Al–7Nb–0.3La	3.290	–	25.61	35.622	−0.670
α–Ti №01–089–3073 ICSD	2.951	4.685	–	183.594	–
β–Ti №01–089–4913 ICSD	3.283	–	–	35.384	–

**Table 8:** True density of Ti–6Al–7Nb–0.3REE (REEs—Y, La, Ce) alloys.

Alloy Composition, wt. %	True Density, g/cm^3^	Std. Dev.
Ti–6Al–7Nb–0.3Y	4.4563	±0.1075
Ti–6Al–7Nb–0.3La	4.8019	±0.0111
Ti–6Al–7Nb–0.3Ce	4.7255	±0.2853

**Table 9:** Microindentation results of Ti–6Al–7Nb–0.3REE (REEs—Y, La, Ce) alloys.

Alloy Composition, wt. %	HV 0.1	HV 0.2	HV 0.5	HV 1	HV 2	HV 5	HV 10
Ti–6Al–7Nb–0.3Y	378 ± 12	384 ± 26	355.7 ± 2.3	344 ± 6	345 ± 6	351 ± 6	343 ± 6
Ti–6Al–7Nb–0.3La	364 ± 28	350 ± 26	322.3 ± 2.3	334 ± 18	313 ± 7	323.7 ± 3.5	317 ± 11
Ti–6Al–7Nb–0.3Ce	436 ± 32	351 ± 10	374 ± 23	376 ± 16	329 ± 9	376 ± 18	372 ± 32

**Table 10:** Nanoindentation results for Ti–6Al–7Nb–0.3REE (REEs—Y, La, Ce) alloys: maximum depth, hardness (H), elastic modulus (E) and elastic recovery (R).

Alloy Composition, wt. %	Load F, mN	Depth, h (nm)	Hardness, H (GPa)	Elastic Modulus, E (GPa)	Elastic Recovery, R (%)
Ti–6Al–7Nb–0.3Y	20	406 ± 20	5.20 ± 0.6	143 ± 8	23.3 ± 1.5
	100	998 ± 31	4.67 ± 0.32	140 ± 5	20.5 ± 1.0
	500	2339 ± 67	4.39 ± 0.30	137 ± 3.2	18.8 ± 0.9
Ti–6Al–7Nb–0.3La	20	422 ± 15	4.83 ± 0.37	137 ± 7	22.2 ± 1.4
	100	1037 ± 27	4.29 ± 0.24	134 ± 6	19.5 ± 1.0
	500	2429 ± 64	4.01 ± 0.23	135 ± 8	17.7 ± 0.7
Ti–6Al–7Nb–0.3Ce	20	401 ± 19	5.40 ± 0.5	149 ± 9	22.9 ± 1.3
	100	974 ± 37	4.90 ± 0.4	150 ± 6	19.9 ± 1.1
	500	2266 ± 60	4.67 ± 0.26	146 ± 5	18.6 ± 0.6

**Table 11:** Control measurements of Wistar rats’ body weights during the in vivo experiments after skin post-implantation of Pure Titanium samples (Control Group), Ti–6Al–7Nb–0.3Y samples (Group 1), Ti–6Al–7Nb–0.3La samples (Group 2), and Ti–6Al–7Nb–0.3Ce samples (Group 3). Median (Q1–Q3).

Group Name and Alloy Composition	Parameter	In Vivo Experimental Stages				
		0 Days	7 Days	14 Days	21 Days	28 Days
Control Group Pure Titanium	Me	412	404	393	394	398
	(Q1–Q3)	390–434	388–439	378–439	382–452	386–398
Group 1 Ti–6Al–7Nb–0.3Y	Me	440	435.5	440	429	424
	(Q1–Q3)	417–461	426–479	411–427	409–435.5	410–434
Group 2 Ti–6Al–7Nb–0.3La	Me	523	528	542	560	556
	(Q1–Q3)	446–574	348–540	496–558	514–584	526–574
Group 3 Ti–6Al–7Nb–0.3Ce	Me	552	565	564	577	560
	(Q1–Q3)	507–591	485–582	499–584	502–588	510–592

**Table 12:** Measurements of Wistar rats’ body temperature, including median (Q1–Q3), during in vivo experiments after skin post-implantation of Pure Titanium samples (Control Group), Ti–6Al–7Nb–0.3Y samples (Group 1), Ti–6Al–7Nb–0.3La samples (Group 2), and Ti–6Al–7Nb–0.3Ce samples (Group 3). Median (Q1–Q3).

Group Name and Alloy Composition	Parameter	In Vivo Experimental Stages				
		0 Days	7 Days	14 Days	21 Days	28 Days
Control Group Pure Titanium	Me	37.3 *	36.35 *	36.65 *	36.5 *	36.2 *
	(Q1–Q3)	37.2–37.6	36.3–36.7	36.2–37	36.3–36.5	35.8–36.2
Group 1 Ti–6Al–7Nb–0.3Y	Me	37.1 *	36.35 *	36.4 *	36.3 *	36.5 *
	(Q1–Q3)	36.65–37.55	36.15–36.65	36.2–36.35	36.05–36.35	36.1–36.5
Group 2 Ti–6Al–7Nb–0.3La	Me	37.7 *	36.65 *	36.5 *	36.65 *	36.8 *
	(Q1–Q3)	37.55–37.95	36.4–36.75	36.4–36.5	36.4–36.9	36.6–37
Group 3 Ti–6Al–7Nb–0.3Ce	Me	37.2 *	36.6 *	36.5 *	36.6 *	36.7 *
	(Q1–Q3)	37.1–37.35	36.45–36.8	36.25–36.95	36.4–36.8	36.5–36.8

**Note:** Values marked with an asterisk indicate difference from pre-operative (duration of 0 days) parameters at *p* < 0.05.

**Table 13:** Measurements of Wistar rats’ local body temperature in the implantation area, including median (Q1–Q3), during in vivo experiments of Pure Titanium samples (Control Group), Ti–6Al–7Nb–0.3Y samples (Group 1), Ti–6Al–7Nb–0.3La samples (Group 2), and Ti–6Al–7Nb–0.3Ce samples (Group 3).

Group Name and Alloy Composition	Parameter	In Vivo Experimental Stages				
		0 Days	7 Days	14 Days	21 Days	28 Days
Control Group Pure Titanium	Me	34.1 *	32.5	32 *	33 *	32.5 *
	(Q1–Q3)	34–34.3	32.1–32.9	31.2–32	32–33	31.8–32.5
Group 1 Ti–6Al–7Nb–0.3Y	Me	33.75	32.1 *	32.6 *	32.1 *	32.8 *
	(Q1–Q3)	33.05–34.2	31.35–33.1	32.15–32.4	31.65–32.1	32.1–32.8
Group 2 Ti–6Al–7Nb–0.3La	Me	34.95	**34.2**	32.0 *	32.5 *	33.0 *
	(Q1–Q3)	34.05–35	33.55–34.85	31–32.8	32–33.8	32.2–33.2
Group 3 Ti–6Al–7Nb–0.3Ce	Me	34.0	33.3	32.55 *	32.9 *	32.7 *
	(Q1–Q3)	33.8–34.2	32.3–34.1	32.05–33.3	31.95–33.1	31.55–33.45

**Note:** Values marked with an asterisk indicate difference from pre-operative (duration of 0 days) parameters at *p* < 0.05; values marked in bold show difference in the indicator in comparison with the Control Group, Group 1, and Group 3 at *p* < 0.05.

**Table 14:** Serum biochemical parameters, including median (Q1–Q3), of Wistar rats’ blood 3 months post-skin implantation of Pure Titanium samples (Control Group), Ti–6Al–7Nb–0.3Y samples (Group 1), Ti–6Al–7Nb–0.3La samples (Group 2), and Ti–6Al–7Nb–0.3Ce samples (Group 3).

Parameter	Control Group Pure Titanium	Group 1 Ti–6Al–7Nb–0.3Y	Group 2 Ti–6Al–7Nb–0.3La	Group 3 Ti–6Al–7Nb–0.3Ce
Total protein, g/L	62 (61–63)	65 (62–67)	66 (63–68)	64 (62–68)
CRP (C-reactive protein), μg/L	4.7 (4.0–5.5)	3.4 (2.8–3.6)	3.6 (2.0–5.5)	7.4 (5.2–8.2)
ALT (alanine aminotransferase), U/L	60 (51–69)	58 (57–63)	**102 (94–111)** ***p* = 0.05**	84 (72–92)
AST (aspartate aminotransferase), U/L	146 (121–167)	160 (143–164)	162 (157–167)	161 (157–167)
LDH (lactate dehydrogenase), U/L	1089 (1009–1250)	948 (906–1072)	**2797 (2335–3352)** ***p* = 0.05**	**2165 (1990–2569)** ***p* = 0.04**
Urea, mmol/L	5.2 (4.7–5.6)	5.8 (5.4–6.0)	5.7 (5.4–5.8)	5.7 (5.3–5.9)
Creatinine, μmol/L	54 (52–57)	57 (56–60)	**61 (60–62)** ***p* = 0.05**	**61 (59–61)** ***p* = 0.04**
Glucose, mmol/L	19.1 (17.1–21.4)	22.1 (21.7–22.8)	16.2 (15.1–16.5)	15.9 (14.7–16.9)
LMMWSs (low- and medium-molecular-weight substances), conv. units	5.80 (5.43–5.98)	6.17 (6.04–6.95)	**6.82 (6.61–** **7.27)** ***p* = 0.05**	**6.86 (6.76–7.35)** ***p* = 0.04**
Catalase activity, %	14.5 (14.1–15.0)	**16.6 (15.6–17.2)** ***p* = 0.05**	17.2 (15.0–19.4)	**17.4 (16.0–19.1)** ***p* = 0.05**

**Note:** Values marked in bold indicate parameters for which the difference from the Control Group reached statistical significance. Specific *p*-values are provided for each such parameter.

## Data Availability

The original contributions presented in this study are included in the article. Further inquiries can be directed at the corresponding author.
